# Synthetic production of prenylated naringenins in yeast using promiscuous microbial prenyltransferases

**DOI:** 10.1016/j.mec.2021.e00169

**Published:** 2021-03-19

**Authors:** Shota Isogai, Nobuyuki Okahashi, Ririka Asama, Tomomi Nakamura, Tomohisa Hasunuma, Fumio Matsuda, Jun Ishii, Akihiko Kondo

**Affiliations:** a Graduate School of Science, Technology and Innovation, Kobe University, 1-1 Rokkodai, Nada, Kobe, 657-8501, Japan; b Technology Research Association of Highly Efficient Gene Design (TRAHED), Kobe, Japan; c Engineering Biology Research Center, Kobe University, 1-1 Rokkodai, Nada, Kobe, 657-8501, Japan; d Department of Bioinformatic Engineering, Graduate School of Information Science and Technology, Osaka University, 1-5 Yamadaoka, Suita, Osaka, 565-0871, Japan; e Department of Chemical Science and Engineering, Graduate School of Engineering, Kobe University, 1-1 Rokkodai, Nada, Kobe, 657-8501, Japan; f Center for Sustainable Resource Science, RIKEN, 1-7-22 Suehiro, Tsurumi, Yokohama, 230-0045, Japan

**Keywords:** Prenylnaringenin, Prenyltransferase, Naringenin, Prenylflavonoids, Yeast

## Abstract

Reconstitution of prenylflavonoids using the flavonoid biosynthetic pathway and prenyltransferases (PTs) in microbes can be a promising attractive alternative to plant-based production or chemical synthesis. Here, we demonstrate that promiscuous microbial PTs can be a substitute for regiospecific but mostly unidentified botanical PTs. To test the prenylations of naringenin, we constructed a yeast strain capable of producing naringenin from l-phenylalanine by genomic integration of six exogenous genes encoding components of the naringenin biosynthetic pathway. Using this platform strain, various microbial PTs were tested for prenylnaringenin production. *In vitro* screening demonstrated that the fungal AnaPT (a member of the tryptophan dimethylallyltransferase family) specifically catalyzed C-3′ prenylation of naringenin, whereas SfN8DT-1, a botanical PT, specifically catalyzed C-8 prenylation. *In vivo*, the naringenin-producing strain expressing the microbial AnaPT exhibited heterologous microbial production of 3′-prenylnaringenin (3′-PN), in contrast to the previously reported *in vivo* production of 8-prenylnaringenin (8-PN) using the botanical SfN8DT-1. These findings provide strategies towards expanding the production of a variety of prenylated compounds, including well-known prenylnaringenins and novel prenylflavonoids. These results also suggest the opportunity for substituting botanical PTs, both known and unidentified, that display relatively strict regiospecificity of the prenyl group transfer.

## Highlights

• Promiscuous microbial prenyltransferases replaced regiospecific botanical enzymes.
• A stable yeast strain that produced naringenin from l-phenylalanine was constructed.
• A fungal prenyltransferase (AnaPT) catalyzed C-3′ prenylation of naringenin.
• AnaPT catalyzed the first microbial production of 3′-prenylnaringenin.
• Microbial prenyltransferases permit the production of various prenylated compounds.

## 1 Introduction

The polyphenolic compounds known as flavonoids constitute a large family of secondary metabolite that are recognized as for their health-promoting properties. To date, over 7,000 flavonoids have been identified from plants; most of these molecules are promising compounds that show a variety of biological activities (Grotewold, 2006). Within this family, prenylflavonoids form a unique subclass of phytochemicals that have one or more prenyl groups, such as dimethylallyl (C_5_) and geranyl (C_10_) moieties, attached to their flavonoid backbone. The structural diversity of prenylflavonoids arises from the addition of varied position, length, and number of prenyl moieties and associated modifications such as cyclization, oxidation, and side chain elongation (Zhao, 2003). Indeed, to date, over 1,000 prenylflavonoids have been isolated from members of the plant kingdom. A number of prenylflavonoids have been shown to possess valuable biological and pharmacological activities, including (for example) anti-oxidant, anti-bacterial, anti-tumor, anti-inflammatory, enzyme inhibitor, and estrogenic activities (Botta et al., 2005; Chen et al., 2014; Terao and Mukai, 2014; Yazaki et al., 2009). Notably, prenylation endows flavonoid backbones with a range of enhanced and distinct biological activities. For instance, prenylation increases the estrogenic activity of naringenin or genistein approximately 100- to 1000-fold (Kretzschmar et al., 2010), while 3-prenylation of luteolin renders the resulting molecule an inhibitor of tyrosinase (Arung et al., 2010).

As noted above, prenylflavonoids have attracted significant attention for use in clinical, research, and industrial applications. However, the limited supply of these compounds prevents their wider use for these purposes; strategies for increasing the availability of prenylflavonoids are strongly desired. Although production of prenylflavonoids relies primarily on isolation from plants or organic synthesis (Yang et al., 2015), plants contain small quantities of these compounds, making extraction and purification arduous and expensive (Grienke et al., 2016; Kuete et al., 2014). Organic synthesis also is a challenge in most cases, given the structural complexity of the compounds of interest (Kawamura et al., 2014; Neves et al., 2011).

**Fig. 1:**
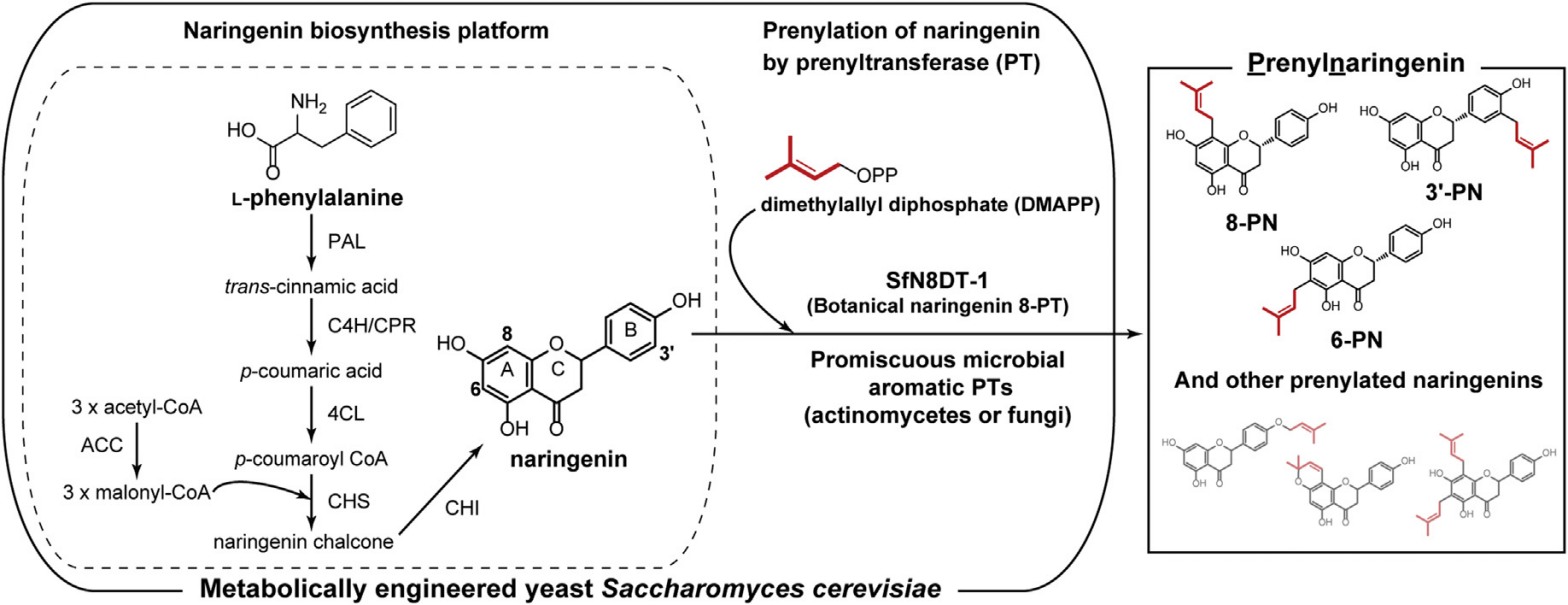
Schematic illustration of *de novo* reconstitution of prenylnaringenin biosynthetic pathway in the yeast *Saccharomyces cerevisiae*. This approach includes two important strategic aspects: a naringenin biosynthesis platform (surrounded by the dashed line) and a prenyltransferase (PT) module for catalyzing the prenylation of the naringenin product. In the naringenin biosynthesis platform, six enzymes serve to produce naringenin from ʟ-phenylalanine through five metabolic reaction steps. Synthesized naringenin then is prenylated by PTs of varying specificities, which catalyze this key step of prenylnaringenin biosynthesis. The botanical naringenin 8-prenyltransferase (SfN8DT-1) and promiscuous microbial (actinomycete and fungal) PTs are used for prenylation of naringenin. Structures of known prenylnaringenins isolated from plants are presented, and include 8-prenylnaringenin (8-PN), 3′-prenylnaringenin (3′-PN), and 6-prenylnaringenin (6-PN). PAL, phenylalanine ammonia lyase; C4H, trans-cinnamate 4-monooxygenase (a cytochrome P450 enzyme); CPR, cytochrome P450 reductase; 4CL, 4-coumaric acid-CoA ligase; CHS, chalcone synthase; CHI, chalcone isomerase.

Reconstitution of prenylflavonoid production in microorganisms using the combination of a flavonoid biosynthetic pathway and a prenyltransferase (PT) can be a promising attractive alternative to plant-based production or chemical synthesis. Recombinant microbes have great potential for cost-effective and selective mass production of prenylflavonoids. *In vitro* biosynthesis of flavonoid backbones and prenyl side chains using appropriate purified enzymes also is expected to reproduce the structural diversity of prenylflavonoids. Naringenin, one of the most basic flavonoid backbones, is derived from l-phenylalanine by a fermentation process; the corresponding biosynthetic pathway is composed of five metabolic steps requiring six enzymes, including a cytochrome P450-mediated reaction (Fig. 1). The naringenin biosynthetic pathway also can contribute to the production of other flavonoids such as isoflavonoids, flavonols, and anthocyanins (Grotewold, 2006). Thus, once a naringenin-producing microbe has been constructed, other related flavonoids can be produced by the introduction of additional modifying enzymes.

In the biosynthesis of prenylflavonoids, the PT is a key enzyme, given that the position of the prenyl group determines the biological activity of the prenylflavonoid (Chen et al., 2014). Three prenylated naringenin derivatives (8-prenylnaringenin (8-PN), 3′-prenylnaringenin (3′-PN), and 6-prenylnaringnin (6-PN)) have been isolated from plants; these compounds represent modifications with dimethylallyl groups at the C-8, C-3′, and C-6 position (respectively) of the backbone (Fig. 1). Despite sharing the same parent backbone, these prenylnaringenins show different biological activities: 8-PN exhibits estrogenic activity (Kretzschmar et al., 2010) and prevents atrophy of disused muscle when provided as a dietary supplement (Mukai et al, 2012, 2016); 3′-PN inhibits the activity of protein tyrosine phosphatase-1B (PTP1B), a target for the treatment of Type 2 diabetes (Cui et al., 2007); and 6-PN is anti-oxidant and preferentially enhances the estrogen 2-hydroxylation pathway (leading to decreased risk for breast cancer) by up-regulating expression of cytochrome P450 1A1 (Wang et al., 2016).

To date, only thirteen flavonoid PTs have been identified in plants; these botanical PTs are membrane bound enzymes that display relatively strict regiospecificity for prenyl group transfer (Akashi et al., 2009; Chen et al., 2013; Li et al., 2014; Sasaki et al, 2008, 2011; Shen et al., 2012; Wang et al., 2014; Yoneyama et al., 2016). The small number of botanical PTs and their characteristic features (membrane-bound nature and strict regiospecificity) limits the microbial production of various prenylflavonoids. One such PT, SfN8DT-1 from *Sophora flavescens*, was characterized as a naringenin PT that catalyzes C-8 prenylation (Sasaki et al., 2008). SfN8DT-1 also catalyzes the prenylation of liquiritigenin and hesperetin; however, this enzyme regiospecifically transfers a prenyl group only onto the C-8 of the A-ring in these flavonoid backbones. PTs that catalyze C-3’ or C-6 prenylation of naringenin have not been identified yet.

In contrast, actinomycetes or fungi employ promiscuous PTs in the biosynthesis of secondary metabolites. Such promiscuous PTs might be alternatives to botanical PTs for the production of prenylflavonoids. There are (to our knowledge) no reports of the endogenous biosynthesis or activity of prenylflavonoids in microorganisms; indeed, under natural conditions, these promiscuous microbial PTs would not encounter flavonoids. The native role of these microbial PTs is to transfer the prenyl group onto the aromatic ring of substrate such as naphthalene-, phenylalanine-, and tryptophan-derived compounds. Nonetheless, these PTs have been also shown to catalyze the *in vitro* prenylation of flavonoids, consistent with the relaxed substrate specificity of microbial PTs (in contrast to botanical PTs) (Kumano et al., 2008; Ozaki et al., 2009; Zhou et al., 2015). Microbial PTs are soluble enzymes and belong to the ABBA prenyltransferase superfamily, which shares a unique protein fold (termed the PT-barrel) comprising five repetitive αββα-motifs. In a barrel fold protein family, TIM-barrel is well known to have the (β/α)_8_-barrel structure widely conserved in many proteins (Gerlt and Raushel, 2003). In contrast to the TIM-barrel that forms an open barrel by the parallel β-strands, the PT barrel forms a central β-barrel fold consisting of ten antiparallel β-strands and contains the active central core with a spacious cavity (Bonitz et al., 2011; Tello et al., 2008). SCO7190(Kumano et al., 2008), NovQ (Ozaki et al., 2009), and NphB (Kumano et al., 2008), which are actinomycete members of this superfamily, have been shown to catalyze the prenylation of flavonoids. Specifically, transgenic plants expressing SCO7190 and NovQ produced prenylflavonoids (6-PN and 6-prenylgenistein) (Sugiyama et al., 2011); NphB purified from *Escherichia coli* provided *in vitro* transfer of a geranyl group onto C5’ of the B-ring of the flavonoid backbone (dihydroxychrysin) (Shindo et al., 2011). Thus, promiscuous PTs from microbes such as actinomycetes and fungi are expected to be active in other microbes, permitting the *in vivo* biosynthesis of prenylflavonoids.

The budding yeast *Saccharomyces cerevisiae* is a leading microorganism for heterologous production of valuable metabolites. The eukaryotic nature of budding yeast is expected to facilitate functional expression of plant-derived genes such as those encoding cytochrome P450 genes (Brown et al., 2015; Koopman et al., 2012; Shin et al., 2012; Vos et al., 2015) and membrane-bound PTs (Sasaki et al., 2008). If microbes like yeast enable the synthetic *in vivo* production of various prenylflavonoids from comparatively inexpensive amino acids as substrate feedstocks, the limited supply of these secondary metabolites could be resolved.

In the present study, we demonstrated the synthetic microbial production of prenylated flavonoids from l-phenylalanine in recombinant yeast strains. Naringenin was targeted as the major flavonoid backbone, and we reconstituted in yeast the naringenin biosynthetic pathway (consisting of five metabolic steps and six enzymes, including a cytochrome P450 reaction). To test the artificial biosynthesis of prenylated naringenin in microbes, a regiospecific botanical PT (SfN8DT-1) and a variety of promiscuous microbial PTs were introduced into the naringenin-producing yeast strain. We successfully obtained two yeast strains, one each producing 8-PN or 3′-PN from l-phenylalanine. The latter strain, which employs a promiscuous microbial PT from *Neosartorya fischeri*, is the first (to our knowledge) demonstration of heterologous microbial production of 3′-PN. This result suggests that microbial PTs with relaxed substrate specificity have great potential to replace regiospecific botanical PTs, most which remain unidentified, and to expand the available position of prenyl moieties in the flavonoid modification step.

## 2 Materials and methods

### 2.1 Chemicals

l-phenylalanine, 2-phenylethanol, *trans*-cinnamic acid, and trifluoroacetic acid (TFA) were purchased from Nacalai Tesque (Kyoto, Japan). *p*-Coumaric acid was obtained from Sigma-Aldrich (St. Louis, MO, USA). Naringenin was obtained from LKT laboratories (St. Paul, MN, USA). 8-Prenylnaringenin (8-PN) and 6-prenylnaringenin (6-PN) were purchased from PhytoLaB (Vestenbergsgreuth, Germany). 3′-Prenylnaringenin (3′-PN) was purchased from Apin Chemicals Ltd. (Abingdon, Oxon, UK). Dimethylallyl pyrophosphate (DMAPP) triammonium salt was obtained from Cayman Chemical (Ann Arbor, MI, USA). HPLC-grade acetonitrile was used for HPLC analysis, and liquid chromatography-mass spectrometry (LC-MS)-grade methanol and acetonitrile were used for LC-MS analysis. All solvents were purchased from Wako Pure Chemical Industries, Ltd. (Osaka, Japan).

### 2.2 Strains and media

*Escherichia coli* DH5α and JM109 (Wako Pure Chemical Industries, Ltd.) were used for gene cloning. Specifically, the latter strain was used for construction of pPT0024-2B. *E. coli* was cultured at 37 ​°C in Luria-Bertani (LB; 10 ​g ​L^−1^ tryptone, 5 ​g ​L^−1^ yeast extract, and 5 ​g ​L^−1^ sodium chloride) broth containing 100 ​μg ​mL^−1^ ampicillin. *Saccharomyces cerevisiae* YPH499 (***MAT*a** *ura3-52 lys2-801 ade2-101 trp1-Δ63 his3- Δ200 leu2- Δ1*) (Sikorski and Hieter, 1989) (Stratagene/Agilent Technologies, Palo Alto, CA, USA) was used as the host strain for heterologous expression. YPDA medium contained 10 ​g ​L^−1^ yeast extract (Nacalai Tesque), 20 ​g ​L^−1^ peptone (BD-Diagnostic Systems, Sparks, MD, USA), 20 ​g ​L^−1^ glucose, and 150 ​mg ​L^−1^ adenine. Synthetic dextrose (SD) medium contained 6.7 ​g ​L^−1^ yeast nitrogen base without amino acids (YNB) (BD-Diagnostic Systems) and 20 ​g ​L^−1^ glucose; SD was supplemented with the appropriate amino acids and nucleotides (40 ​mg ​L^−1^ adenine, 20 ​mg ​L^−1^ histidine, 60 ​mg ​L^−1^ leucine, 20 ​mg ​L^−1^ lysine, 40 ​mg ​L^−1^ tryptophan, and 20 ​mg ​L^−1^ uracil) to provide for the relevant auxotrophies. All six amino acids and nucleotides were supplemented for minimal medium. For use in cultures for metabolite production, SD medium or YPDA medium was further supplemented with 10 ​mM ʟ-phenylalanine.

### 2.3 Genes

The genes used in this study are listed in Supplementary Table S1. Genomic DNA from *S. cerevisiae* YPH499 was used for cloning of the *ACC1* gene. Full-length Arabidopsis cDNA clones were used as the template DNAs for the PCR amplification of naringenin biosynthetic genes. The cDNA clones (and corresponding genes) used in this study were as follows: pda01171 (*AtPAL1*), pda03082 (*AtC4H*), pda02355 (*AtCPR1*), pda05828 (*At4CL3*), and pda05357 (*AtCHS3*). These clones were obtained from RIKEN BRC through the National Bio-Resource project of MEXT, Japan (Seki et al, 1998, 2002). Note that pda05828 carries only a partial open reading frame (ORF); therefore, a codon-optimized (“co”) full-length *4CL3* ORF, designated *coAt4CL3*, was synthesized separately. Several genes used in this study were optimized for *S. cerevisiae* codon usage. Specifically, the *At4CL3* and *AtCHI1* genes were optimized using the OptimumGene™ Gene Design system; the codon-optimized *coAt4CL3* and *coAtCHI1* genes were synthesized by the GenScript Gene Synthesis service (GenScript, Piscataway, NJ, USA) and sub-cloned into pUC57 (GenScript). Codon optimization of other genes (see below) was conducted using GeneArt® GeneOptimizer software (Life Technologies/Thermo Fisher Scientific, San Jose, CA, USA). The codon-optimized *cdpNPT* gene (*coCdpNPT*) (*Aspergillus fumigatus*) was synthesized using the GeneArt® Gene Synthesis service and sub-cloned into the pMX vector (Life Technologies/Thermo Fisher Scientific). Other codon-optimized genes, including *coAtCHS3* (Fragment-1 and -2) (*Arabidopsis thaliana*), *coSCO7190* (*Streptomyces coelicolor*), *coSfN8DT-1* (Fragment-1 and -2) (*S. flavescens*), *coNovQ* (*Actinoalloteichus cyanogriseus*), *co5-DMATS* (*Aspergillus clavatus*), *co6-DMATS* (*Streptomyces ambofaciens*), *co7-DMATS* (*A. fumigatus*), *coAnaPT* (*Neosartorya fischeri*), *coCdpC3PT* (*N. fischeri*), *coFgaPT2* (*A. fumigatus*), and *coFtmPT1* (*A. fumigatus*), were prepared using the GeneArt® Strings™ DNA fragments service. These DNA fragments were used as template DNAs for PCR amplification.

### 2.4 Construction of plasmids

KOD -Plus- Neo (TOYOBO, Osaka, Japan) and PrimeSTAR HS DNA polymerase (TaKaRa Bio, Shiga, Japan) were used for PCR amplification. The sets of forward and reverse primers are listed in Supplementary Table S2. The scheme of plasmid construction is illustrated in Supplementary Figs. S1 and S2. The pPF42x series are multi-copy plasmids harboring naringenin biosynthetic genes. The *AtPAL1*, *AtC4H*, and *AtCHS3* genes were amplified from the Arabidopsis full-length cDNAs (pda01171, pda03082, and pda05357, respectively) and introduced between the *Avr*II and *Fse*I sites of the pATP424, pATP425, and pATP426 vectors (Ishii et al., 2014) (respectively) using the In-Fusion® HD Cloning Kit (Takara Bio). PCR amplification was performed using the Arabidopsis full-length cDNA pda02355 as the template to generate a DNA fragment containing *AtCPR1*; this fragment was employed for In-Fusion cloning between the *Sal*I and *Not*I sites of pATP425, yielding plasmid pPF005. The sequence-validated *coAt4CL3* gene was digested with *Sal*I and *Not*I; the resulting *coAt4CL3* DNA fragment was ligated into the same restriction sites of plasmid pPF4240, yielding pPF4241. An equivalent set of reactions was used to insert the *coAtCHI1* gene into pATP426 harboring the *AtCHS3* gene, yielding plasmid pPF4262. The *coAtCHS3* gene was amplified by overlap PCR using Fragment-1 (1–700 bp) and −2 (601–1188 bp) and exchanged for the *AtCHS3* gene of pPF4262, yielding plasmid pPF4263.

The pPF00x series are genome integration plasmids and the strategy of construction is shown in Supplementary Fig. S2. A fragment including T_ADH1_-*AtPAL1*-P_ADH1_-T_TDH3_- *coAt4CL3*-P_TDH3_ was prepared by the *Xho*I-*Spe*I digestion of pPF4242 and ligated into the similarly digested pRS404red vector (a pRS404-based vector in which the marker region was modified to enable integration into the *trp1-*Δ*63* locus; under preparation for submission elsewhere), yielding plasmid pPF0041. pPF425 was digested with *Fse*I and *Not*I and the *AtC4H*-P_ADH1_-T_TDH3_- *At4CPR1* DNA fragment was obtained. This fragment was ligated into the similarly digested pATP405, yielding plasmid pPF005. Construction of pPF006 was accomplished by the removal of the 2μ origin from pPF4263 by *Aat*II digestion following by self-ligation.

The strategy for constructing PT-expressing plasmids is shown Supplementary Figs. S1 and S2. At first, multi-copy expression plasmids were constructed (Supplementary Fig. S1). Construction of pACC was performed by PCR amplification of the *ACC1* gene using *S. cerevisiae* genomic DNA as the template; the resulting amplicon was cloned between the *Avr*II and *Fse*I sites of pATP422 by In-Fusion cloning. The *coSCO7190* and *coNovQ* genes were amplified using the respective synthesized DNA fragments, while *coSfN8DT-1* was prepared by the overlap PCR using Fragment-1 (1–660 bp) and −2 (601–1233 bp). These DNA fragments were introduced into the *Mlu*I site of pACC using the In-Fusion® HD Cloning Kit, yielding plasmids pACC-PT1 (harboring *coSCO7190*), pACC-PT2 (*coSfN8DT-1*), and pACC-PT3 (*coNovQ*). However, these plasmids were not used in further experiments because these multi-copy plasmids were not stably maintained. Instead, genome integration plasmids were constructed for expression of the PTs. DNA fragments including T_ADH1_-*ACC1*-P_ADH1_-T_TDH3_-*PT*-P_TDH3_ were prepared by the *Xho*I-*Sac*I digestion of pACC-PT1, -PT2, and -PT3. These DNA fragments were ligated (separately) into pATP402, yielding plasmids pPT021 (harboring *coSCO7190*), pPT0022 (*coSfN8DT-1*), and pPT0023 (*coNovQ*). Other codon-optimized PT genes were amplified using artificial synthesized DNA fragments and exchanged for the *coSCO7190* gene of pPT0021, yielding plasmids pPT0024 (*co5-DMATS*), pPT0025 (*co6-DMATS*), pPT0026 (*co7-DMATS*), pPT0027 (*coAnaPT*), pPT0028 (*coCdpC3PT*), pPT0029 (*coCdpNPT*), pPT002A (*coFgaPT2*), and pPT002B (*coFtmPT1*).

### 2.5 Yeast transformation

Transformation of *S. cerevisiae* YPH499 was performed using the lithium-acetate method (Gietz et al., 1992). The genomic integration plasmids were linearized before transformation, using *Hpa*I for pPF0042 and pPF005, *Eco*RV for pPF0063, and *Afl*II for pPT002x (where x ​= ​1–9, A, or B). The maintenance of genome-integrated plasmids in transformants was checked by colony-direct PCR using KOD FXneo (TOYOBO) and four independent transformants were utilized for the subsequent experiments. Supplementary Fig. S3 shows the strains constructed in this study and descriptions of these strains.

### 2.6 Cultivation of transformants and extraction of metabolites

The transformants were cultured overnight at 30 ​°C in SD selection medium (for strains harboring multi-copy plasmids) or in YPDA medium (for strains harboring genome-integration plasmids). The pre-culture was inoculated into 40 ​mL of the production medium (SD selection medium or YPDA medium containing 10 ​mM ʟ-phenylalanine) in a 100-mL flask at an initial optical density at 660 ​nm (OD_660_) of 0.03. After cultivation at 30 ​°C under rotary shaking of 150 ​revolutions per minute (rpm) for four days, the yeast cells in 30 ​mL of culture broth were harvested by centrifuging at 4,800×*g* for 5 ​min at room temperature. The resulting yeast cell pellet and the supernatant were separated and stored at −30 ​°C and −80 ​°C, respectively. At the same time, the yeast cells in 1 ​mL of the culture broth were collected by centrifuging at 20,630×*g* for 5 ​min. The resulting cell pellet was lyophilized, and the dry cell weight (DCW) was measured.

Next, the collected yeast cells obtained from the 30-mL aliquot of culture broth were suspended in 3 ​mL of Milli-Q and combined with an equal volume (approximately total 6 ​mL) of glass beads. The yeast cells were disrupted by shaking at 1,500 ​rpm for 10 ​min using a ShakeMaster Neo (Biomedical Science, Tokyo, Japan). The disrupted yeast cells were extracted with 6 ​mL of ethyl acetate. After centrifugation at 9,400×*g* for 1 ​min, 4 ​mL of the organic fraction were collected. Extraction was carried out twice and the resulting collected ethyl acetate solution (approximately 8 ​mL) was evaporated using a centrifugal evaporator at 30 ​°C for 60 ​min. The residue was resuspended in 0.5 ​mL of methanol, and the resulting methanol suspension then was filtered using a Millex-LH 0.45-mm (PTFE) filter (Millipore, Billerica, MA, USA). The resulting filtrates were stored at −30 ​°C until analysis.

For prenylnaringenin production experiments, 300 ​μL of the supernatant were extracted with 600 ​μL of ethyl acetate. An aliquot (400 ​μL) of the resulting ethyl acetate layer was concentrated *in vacuo* and then resuspended in 200 ​μL of methanol. The resulting methanol solution was centrifuged at 20,630×*g* for 5 ​min before analysis to remove any insoluble components.

### 2.7 HPLC and LC-MS analysis of metabolites produced by yeast transformants

The amounts of l-phenylalanine and 2-phenylethanol in the supernatant and 2-phenylethanol in the cell extract were measured using a HPLC system. Prior to HPLC analysis, the supernatant was diluted five-fold with 25% methanol and then filtered using a Mini-UniPrep™ Syringeless Filter 0.45-μm PTFE membrane (Whatman/GE Healthcare, Marlborough, MA, USA). The resulting 20% methanol solution and the cell extract were analyzed (separately) on a Prominence HPLC system (Shimadzu) equipped with a COSMOSIL _5_C_18_-MSII column (4.6 ​× ​150 ​mm, 5 ​μm; column temperature, 30 ​°C; Nacalai Tesque) under the following conditions: mobile phase A, water ​+ ​0.1% TFA; mobile phase B, acetonitrile ​+ ​0.1% TFA; 10–50% B over 24 ​min, 50–98% B over 1 ​min, 98% B for 5 ​min, and then 10% B for 15 ​min; at a flow rate of 1.0 ​mL ​min^−1^. The metabolites were monitored at 215 ​nm using an SPD-20A photodiode array (Shimadzu). For quantitative analysis, authentic reference samples of l-phenylalanine and 2-phenylethanol were used as external standards.

For LC-MS analysis, the supernatant and the cell extract (separately) were diluted with methanol to appropriate concentrations. After the insoluble components were removed by centrifugation, each sample was analyzed using an LCMS-2020 system (Shimadzu) on a COSMOSIL _5_C_18_-MSII column (2.0 ​× ​150 ​mm, 5 ​μm; column temperature, 30 ​°C; Nacalai Tesque), at a flow rate of 0.2 ​mL ​min^−1^. Gradient elution was performed under the following conditions: 20%–60% B over 19 ​min, 60%–98% B over 1 ​min, 98% B for 5 ​min, and then 20% B for 15 ​min (mobile phase A: water ​+ ​0.1% acetic acid; mobile phase B: acetonitrile ​+ ​0.1% acetic acid). MS analysis was performed using electro-spray ionization in the negative ion mode. The amount of product was calculated from a standard curve, which was obtained from the peak area of authentic reference samples using the selected ion monitoring (SIM) mode: *m/z* 147.1 [M-H]^-^ for *trans*-cinnamic acid, *m/z* 163.1 [M-H]^-^ for *p*-coumaric acid, and *m/z* 271.1 [M-H]^-^ for naringenin.

The LC-MS analysis of the metabolites produced by the PT-expressing strains (YPNG008-010 and YPNG015-022) was performed as follows. The supernatant extract and the cell extract were prepared as described above (“*Cultivation of transformants and extraction of metabolites*”). If needed, the extracts were diluted with methanol to appropriate concentrations. For quantification of *trans*-cinnamic acid, *p*-coumaric acid, and naringenin, the extract samples of the supernatant and cell were analyzed using the LCMS-2020 under the conditions described above. Prenylated compounds in the cell extracts were analyzed using the LCMS-2020 system (Shimadzu) equipped with a COSMOSIL _5_C_18_-MSII column (2.0 ​× ​150 ​mm, 5 ​μm; column temperature, 30 ​°C; Nacalai Tesque) at a flow rate of 0.2 ​mL ​min^−1^. Gradient elution was carried out under the following conditions: 20%–80% B over 34 ​min, 80%–98% B over 1 ​min, 98% B for 5 ​min, and then 20% B for 15 ​min (mobile phase A: water ​+ ​0.1% acetic acid; mobile phase B: acetonitrile ​+ ​0.1% acetic acid). Detection was carried out in SIM mode, with *m/z* 339.2 [M-H]^-^ for prenylnaringenin. The identity of prenylated compounds were confirmed by comparing the mass spectrum and the retention time of each sample with authentic 8-PN, 3′-PN, and 6-PN. For quantitative analysis, a standard curve was generated using the peak area of MS chromatograms corresponding to 8-PN, 3′-PN, and 6-PN; the amount of each product then was calculated from the respective standard curve.

### 2.8 Prenylation assay of PTs with naringenin and LC-MS analysis of reaction products

YPNG007-009 and YPNG015-018 were grown overnight in YPDA medium at 30 ​°C and then harvested. The collected yeast cells were suspended in a buffer containing 50 ​mM Tris-HCl (pH 7.5) and 100 ​mM NaCl, and the suspension was adjusted to an OD_660_ ​= ​50 with the same buffer. An aliquot (250 ​μL) of the cell suspension was disrupted by vortexing with glass beads. After removal of disrupted cell debris by centrifugation at 20,630×*g* for 5 ​min, 50 ​μL of the cell lysate were incubated with 0.2 ​mM naringenin and 0.2 ​mM DMAPP. The YPNG008-010 ​cell lysate was adjusted to 5 ​mM MgCl_2_–6H_2_O because SfN8DT-1 requires Mg^2+^ ion for enzyme activity. Similarly, the YPNG015-022 ​cell lysate was adjusted to 10 ​mM CaCl_2_–2H_2_O because Ca^2+^ ions enhance the enzyme activity of DMATS-family PTs. The enzyme reactions were carried out at 30 ​°C for 6 ​h. The reaction products were extracted with 200 ​μL of ethyl acetate. An aliquot (150 ​μL) of the resulting organic phase was concentrated *in vacuo*, and the residue then was dissolved in 50 ​μL of methanol. The resulting methanol solution was analyzed directly using an LCMS-2020 system on a COSMOSIL _5_C_18_-MSII column (2.0 ​× ​150 ​mm, 5 ​μm; column temperature, 30 ​°C; Nacalai Tesque) at a flow rate of 0.2 ​mL ​min^−1^. Gradient elution was performed under the following conditions: 20%–98% B over 34 ​min, 98% B for 5 ​min, and then 20% B for 15 ​min (mobile phase A: water ​+ ​0.1% acetic acid; mobile phase B: acetonitrile ​+ ​0.1% acetic acid). Detection was carried out in SIM mode, using *m/z* 271.1 [M-H]^-^ for naringenin and *m/z* 339.2 [M-H]^-^ for prenylnaringenin.

### 2.9 LC-quadrupole time of flight (QTOF)/MS analysis

LC-QTOF/MS analysis was performed by Nexera X2 (Shimadzu) coupled with LCMS-9030 (Shimadzu). LC conditions were identical to that of LC-MS analysis. LCMS-9030 was operated under the following conditions; ionization, electrospray ionization; nebulizer gas flow, 2.0 ​mL ​min^−1^; heating gas flow, 10 ​L/min; interface temperature, 300 ​°C; drying gas flow, 10 ​L/min; desolvent line temperature, 300 ​°C; heat block temperature, 400 ​°C; MS1 scan range, 70–1000; MS2 scan range, 70–350; precursor *m/z*, 339.1; Q1 resolution, low; collision energy, 35; collision energy spread, 17; collision gas, argon.

## 3 Results

### 3.1 Construction of naringenin-producing yeast using multi-copy plasmids

To make a shared platform strain for prenylnaringenin production, *de novo* reconstitution of the naringenin biosynthetic pathway in the yeast *S. cerevisiae* (Fig. 1) was tested in reference to the previous report (Koopman et al., 2012). This pathway involves five metabolic reactions from l-phenylalanine to naringenin and is catalyzed by six enzymes: phenylalanine ammonia lyase (PAL), *trans*-cinnamate 4-monooxygenase (C4H, a cytochrome P450 enzyme), cytochrome P450 reductase (CPR), 4-coumaric acid-CoA ligase (4CL), chalcone synthase (CHS), and chalcone isomerase (CHI) (Fig. 1). All six naringenin biosynthetic genes were obtained from *Arabidopsis thaliana* (*AtPAL1, AtC4H, AtCPR1, At4CL3, AtCHS3*, and *AtCHI1*, respectively), which had been reported to the successful production of naringenin in *S. cerevisiae* (Koopman et al., 2012; Levisson et al., 2019) (Supplementary Table S1). The full-length cDNA clones of *A. thaliana* were obtained from RIKEN BRC (Seki et al, 1998, 2002) and were used for subcloning of the selected genes. Among them, the *At4CL3* cDNA (pda05828) harbored a partial ORF, so a codon-optimized full-length *4CL3*, designated *coAt4CL3*, was synthesized separately.

These six genes were subcloned into pATP42x series plasmids, which are multi-copy yeast expression vectors for concurrent expression of multiple (up to three) genes; these episomes harbor strong constitutive *ADH1*, *TDH3*, and *PGK1* promoters and different selectable markers (Ishii et al., 2014). The plasmids carrying the six genes were divided into three sets, as follows: *AtPAL1* and *coAt4CL3* into pATP424 (yielding pPF4240 (*AtPAL1* only) and pPF4241 (*AtPAL1* and *coAt4CL3*)), *AtC4H* and *AtCPR1* into pATP425 (yielding pPF425), and *AtCHS3* or *coAtCHS3* and *coAtCHI1* into pATP426 (yielding pPF4262 (*AtCHS3* and *coAtCHI1*) and pPF4263 (*coAtCHS3* and *coAtCHI1*)) (Table 1 and Supplementary Fig. S1). Codon-optimized *AtCHS3* and *AtCHI1* genes also were synthesized to facilitate production of naringenin in *S. cerevisiae*.

**Table 1:** Plasmids used in this study.

Plasmids	Description	Reference
Expression vector		
pATP422	Multi-copy vector (2μ origin), ADE2 marker, T_ADH1_–P_ADH1_, P_TDH3_–T_TDH3_ and P_PGK1_–T_PGK1_	Ishii et al. (2014)
pATP424	Multi-copy vector (2μ origin), TRP1 marker, T_ADH1_–P_ADH1_, P_TDH3_–T_TDH3_ and P_PGK1_–T_PGK1_	Ishii et al. (2014)
pATP425	Multi-copy vector (2μ origin), LEU2 marker, T_ADH1_–P_ADH1_, P_TDH3_–T_TDH3_ and P_PGK1_–T_PGK1_	Ishii et al. (2014)
pATP426	Multi-copy vector (2μ origin), URA3 marker, T_ADH1_–P_ADH1_, P_TDH3_–T_TDH3_ and P_PGK1_–T_PGK1_	Ishii et al. (2014)
pATP402	Integration vector, ADE2 marker, T_ADH1_–P_ADH1_, P_TDH3_–T_TDH3_ and P_PGK1_–T_PGK1_	Ishii et al. (2014)
pRS404red	Integration vector (pRS404-based marker region modified vector), enabling integration into *trp1*-Δ*63* locus	Unpublished
pATP405	Integration vector, LEU2 marker, T_ADH1_–P_ADH1_, P_TDH3_–T_TDH3_ and P_PGK1_–T_PGK1_	Ishii et al. (2014)
pATP406	Integration vector, URA3 marker, T_ADH1_–P_ADH1_, P_TDH3_–T_TDH3_ and P_PGK1_–T_PGK1_	Ishii et al. (2014)
Custom synthesized plasmids harboring codon-optimized gene		
pUC57_coAt4CL3	*coAt4CL3* is cloned in pUC57 (the GenScript gene synthesis service)	This study
pUC57_coAtCHI1	*coAtCHI1* is cloned in pUC57 (the GenScript gene synthesis service)	This study
pMX_coCdpNPT	*coCdpNPT* is cloned in pMX (GeneArt® Gene Synthesis service)	This study
Multi-copy plasmids (2μ origen)		
pACC	pATP422, T_ADH1_–*ACC1*–P_ADH1_	This study
pACC-PT1	pATP422, T_ADH1_–*ACC1*–P_ADH1_ and P_TDH3_–*coSCO7190*–T_TDH3_	This study
pACC-PT2	pATP422, T_ADH1_–*ACC1*–P_ADH1_ and P_TDH3_–*coSfN8DT-1*–T_TDH3_	This study
pACC-PT3	pATP422, T_ADH1_–*ACC1*–P_ADH1_ and P_TDH3_–*coNovQ*–T_TDH3_	This study
pPF4240	pATP424, T_ADH1_–*AtPAL1*–P_ADH1_	This study
pPF4241	pATP424, T_ADH1_–*AtPAL1*–P_ADH1_ and P_TDH3_–*coAt4CL3*–T_TDH3_	This study
pPF425	pATP425, T_ADH1_–*AtC4H*–P_ADH1_ and P_TDH3_–*AtCPR1*–T_TDH3_	This study
pPF4262	pATP426, T_ADH1_–*AtCHS3*–P_ADH1_ and P_TDH3_–*coAtCHI1*–T_TDH3_	This study
pPF4263	pATP426, T_ADH1_–*coAtCHS3*-P_ADH1_ and P_TDH3_–*coAtCHI1*–T_TDH3_	This study
Genome integration plasmids		
pPT0021	pATP402, T_ADH1_–*ACC1*–P_ADH1_ and P_TDH3_–*coSCO7190*–T_TDH3_	This study
pPT0022	pATP402, T_ADH1_–*ACC1*–P_ADH1_ and P_TDH3_–*coSfN8DT-1*–T_TDH3_	This study
pPT0023	pATP402, T_ADH1_–*ACC1*–P_ADH1_ and P_TDH3_–*coNovQ*–T_TDH3_	This study
pPT0024	pATP402, T_ADH1_–*ACC1*–P_ADH1_ and P_TDH3_–*co5-DMATS*–T_TDH3_	This study
pPT0025	pATP402, T_ADH1_–*ACC1*–P_ADH1_ and P_TDH3_–*co6-DMATS*–T_TDH3_	This study
pPT0026	pATP402, T_ADH1_–*ACC1*–P_ADH1_ and P_TDH3_–*co7-DMATS*–T_TDH3_	This study
pPT0027	pATP402, T_ADH1_–*ACC1*–P_ADH1_ and P_TDH3_–*coAnaPT*–T_TDH3_	This study
pPT0028	pATP402, T_ADH1_–*ACC1*–P_ADH1_ and P_TDH3_–*coCdpC3PT*–T_TDH3_	This study
pPT0029	pATP402, T_ADH1_–*ACC1*–P_ADH1_ and P_TDH3_–*coCdpNPT*–T_TDH3_	This study
pPT002A	pATP402, T_ADH1_–*ACC1*–P_ADH1_ and P_TDH3_–*coFgaPT2*–T_TDH3_	This study
pPT002B	pATP402, T_ADH1_–*ACC1*–P_ADH1_ and P_TDH3_–*coFtmPT1*–T_TDH3_	This study
pPF0041	pRS404red, T_ADH1_–*AtPAL1*–P_ADH1_ and P_TDH3_–*coAt4CL3*–T_TDH3_	This study
pPF005	pATP405, T_ADH1_–*AtC4H*–P_ADH1_ and P_TDH3_–*AtCPR1*–T_TDH3_	This study
pPF0063	pATP406, T_ADH1_–*coAtCHS3*–P_ADH1_ and P_TDH3_–*coAtCHI1*–T_TDH3_	This study

co: codon optimized.

**Fig. 2:**
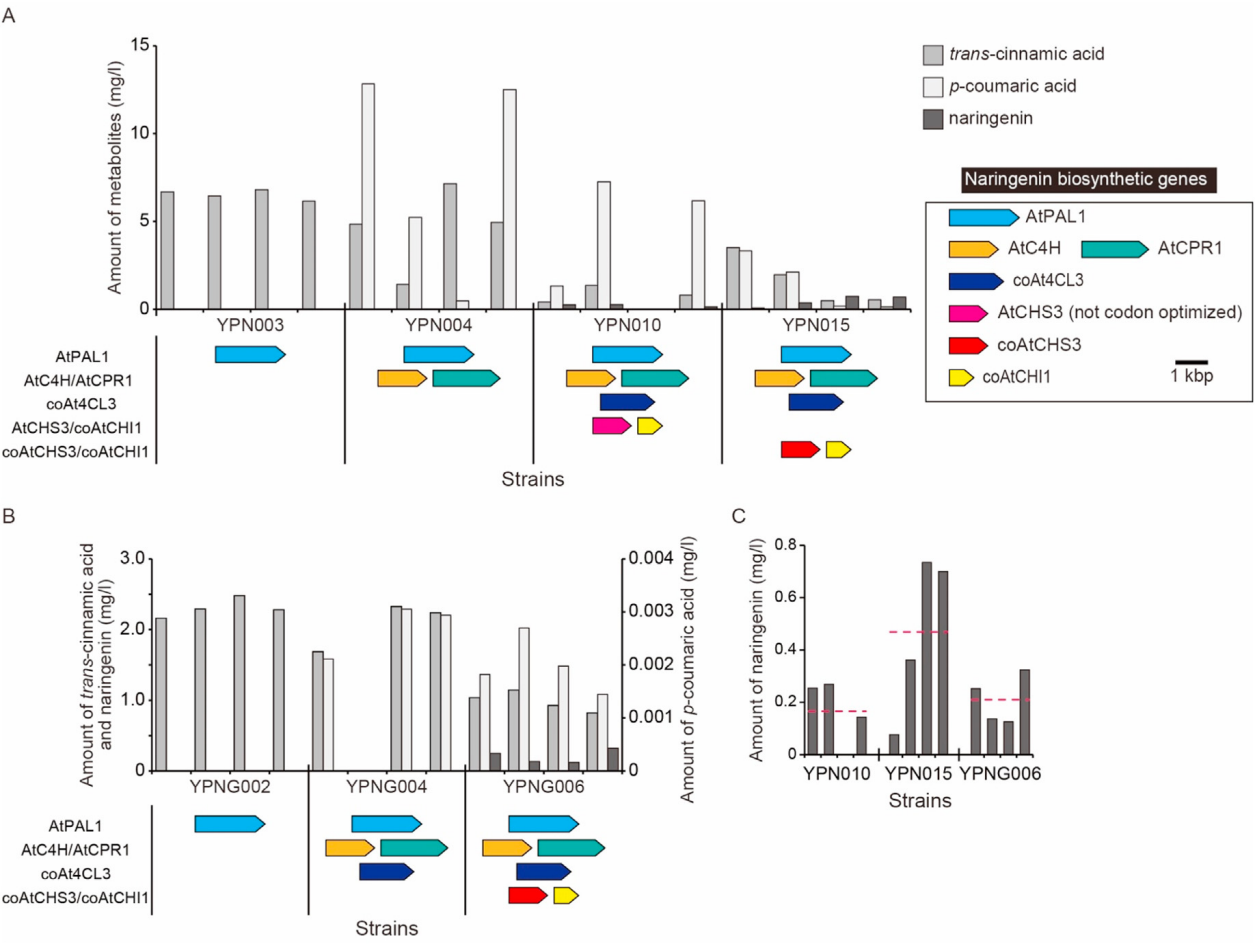
Production of naringenin and intermediates using a naringenin-producing yeast platform. Engineered yeasts (characterized as four separate transformant colonies each) were cultured in synthetic minimal SD selection medium (for strains harboring multi-copy plasmids) or rich YPDA medium (for strains harboring genomically integrated plasmids) containing 10 ​mM ʟ-phenylalanine for 96 ​h; internal metabolites then were extracted. The amounts of naringenin and its intermediates (trans-cinnamic acid and *p*-coumaric acid) were analyzed by LC-MS. Colored arrows shown under each bar graph indicate gene(s) harbored by each strain. (A) Engineered yeast strains harboring naringenin biosynthetic genes on multi-copy plasmid(s). (B) Engineered yeast strains harboring naringenin biosynthetic genes on plasmids integrated into the genome. Left axis indicates the amounts of *trans*-cinnamic acid and naringenin; right axis indicates the amount of *p*-coumaric acid. (C) Comparison of naringenin production among YPN010 (multi-copy plasmids, using non-codon-optimized *AtCHS3* gene), YPN015 (multi-copy plasmids, using codon-optimized *coAtCHS3* gene), and YPNG006 (genomically integrated plasmid, using codon-optimized *coAtCHS3* gene). Dashed bars indicate the mean naringenin amounts.

Yeast strains harboring these multi-copy plasmids were designated YPN003, YPN004, YPN010, and YPN015, corresponding to YPH499 with pPF4240, pPF4240/pPF425, pPF4240/pPF425/pPF4262, and pPF4240/pPF425/pPF4263, respectively (Table 2); control strains consisting of YPH499 harboring empty vector(s) (designated YPN001, YPN002, and YPN006) were constructed in parallel (Table 2). All of these yeast strains were cultured in synthetic minimal medium containing 10 ​mM ʟ-phenylalanine, and the produced metabolites then were extracted and analyzed. Fig. 2A shows the amounts of the target metabolite (naringenin) and intermediates (*trans*-cinnamic acid and *p*-coumaric acid) detected in the various strains. The negative control strains (YPN001, YPN002, and YPN006) produced no naringenin or intermediates (data not shown). YPN003 (*AtPAL1* only) reproducibly generated *trans*-cinnamic acid (Fig. 2A). YPN004 (*AtPAL1*, *AtC4H*, and *AtCPR1*) synthesized both *trans*-cinnamic acid and *p*-coumaric acid, consistent with the corresponding genotypes of the inserted gene(s), although the amounts of *trans*-cinnamic acid and *p*-coumaric acid varied in different colonies (transformants). This observation indicated that the cytochrome P450 reaction proceeded successfully in *S. cerevisiae*. Both YPN010 (all six genes; including non-codon-optimized *AtCHS3*) and YPN015 (all six genes; including codon-optimized *coAtCHS3*) produced naringenin as expected, although the amounts of the measured compounds (*trans*-cinnamic acid, *p*-coumaric acid, and naringenin) further varied among the different colonies. Interestingly, YPN015, which harbors the codon-optimized *coAtCHS3* gene, accumulated naringenin to nominally higher levels than did YPN010. These results indicated that *de novo* reconstitution of the naringenin biosynthetic pathway in yeast was possible using *A. thaliana*-derived genes, as previously demonstrated (Koopman et al., 2012). Notably, we found that codon optimization of the *AtCHS3* gene was effective in increasing naringenin production.

**Table 2:** Yeast strains constructed in this study.

Strains	Description	Source or Reference
YPH499	Host strain for heterologous expression (***MAT*a***ura3-52 lys2-801 ade2-101 trp1-*Δ*63 his3-*Δ*200 leu2-*Δ*1*)	Stratagene, Sikorski and Hieter (1989)
**Strains harboring multi-copy plasmid(s)**		
YPN001	pATP424	This study
YPN002	pATP424, pATP425	This study
YPN003	pPF4240	This study
YPN004	pPF4240, pPF425	This study
YPN006	pATP424, pATP425, pATP426	This study
YPN010	pPF4241, pPF425, pPF4262	This study
YPN015	pPF4241, pPF425, pPF4263	This study
**Strains harboring integration plasmid(s)**		
YPNG001	pRS404red	This study
YPNG002	pPF0041	This study
YPNG003	pRS404red, pATP405	This study
YPNG004	pPF0041, pPF005	This study
YPNG005	pRS404red, pATP405, pATP406	This study
YPNG006	pPF0041, pPF005, pPF0063	This study
YPNG007	pRS404red, pATP405, pATP406, pATP402	This study
YPNG008	YPNG006 harboring pPT0021	This study
YPNG009	YPNG006 harboring pPT0022	This study
YPNG010	YPNG006 harboring pPT0023	This study
YPNG015	YPNG006 harboring pPT0024	This study
YPNG016	YPNG006 harboring pPT0025	This study
YPNG017	YPNG006 harboring pPT0026	This study
YPNG018	YPNG006 harboring pPT0027	This study
YPNG019	YPNG006 harboring pPT0028	This study
YPNG020	YPNG006 harboring pPT0029	This study
YPNG021	YPNG006 harboring pPT002A	This study
YPNG022	YPNG006 harboring pPT002B	This study

### 3.2 Construction of naringenin-producing yeast platform by genome integration

Although *de novo* reconstitution of naringenin biosynthesis in yeast succeeded, the amount of produced naringenin varied substantially and showed low reproducibility among different colonies of YPN015 transformants (*n* ​= ​4) (Fig. 2A). This variability probably was due to the unstable maintenance of multiple plasmids caused by the use of the same plasmid origin (2*μ*) (Futcher and Cox, 1984). To overcome this issue, genome-integration plasmids, designated pPF0041 (*AtPAL1* and *coAt4CL3*), pPF005 (*AtC4H* and *AtCPR1*), and pPF0063 (*coAtCHS3* and *coAtCHI1*), were constructed (Supplementary Table S2 and Supplementary Fig. S2). Yeast strains harboring genomically integrated plasmids were designated as YPNG002, YPNG004, and YPNG006 (YPH499 with integrated pPF0041, pPF0041/pPF005, and pPF0041/pPF005/pPF0063, respectively) (Table 2). Control strains harboring genomically integrated empty vector(s), designated YPNG001, YPNG003, and YPNG005 (Table 2), were constructed in parallel. All of these strains were grown in YPDA rich medium containing 10 ​mM ʟ-phenylalanine, and the produced metabolites then were extracted and analyzed.

The negative control strains (YPNG001, YPNG003, and YPNG005) produced no naringenin or intermediates (data not shown). As shown in Fig. 2B, YPNG002 synthesized comparatively less *trans*-cinnamic acid (2.30 ​± ​0.113 ​mg ​L^−1^) (*n* ​= ​4) than YPN003 (6.52 ​± ​0.248 ​mg ​L^−1^) (*n* ​= ​4), presumably due to the lower copy number in the strain carrying the single-copy genomic integration. Similarly, while YPNG004 produced both intermediates (*trans*-cinnamic acid and *p*-coumaric acid) at similar amounts (2.08 ​± ​0.283 ​mg ​L^−1^ and 2.70 ​± ​0.419 ​mg ​L^−1^, respectively) (*n* ​= ​3; one colony was omitted due to clearly impaired performance), the levels were nominally lower than those obtained with YPN004 (4.58 ​± ​2.05 ​mg ​L^−1^ and 7.76 ​± ​5.19 ​mg ​L^−1^, respectively) (*n* ​= ​4). However, the variations in the amounts of these intermediates among different colonies were considerably decreased in YPNG004, presumably because the genomically integrated gene expression cassettes stabilized the output phenotypes. Lastly, although YPNG006 (expressing all six genes, including codon-optimized *coAtCHS3*, from genomically integrated plasmids) also showed nominally lower naringenin production (0.210 ​± ​0.0824 ​mg ​L^−1^) (*n* ​= ​4) than did YPN015 (harboring multi-copy plasmids) (0.468 ​± ​0.269 ​mg ​L^−1^) (*n* ​= ​4) (Fig. 2C), the clonal variation among different colonies was much improved (Fig. 2B and C). Therefore, we decided to use YPNG006 as the naringenin biosynthesis platform strain because of its superior reproducibility.

### 3.3 Heterologous production of 8-prenylnaringenin (8-PN) in yeast using naringenin biosynthesis platform and regiospecific botanical prenyltransferase (SfN8DT-1)

Heterologous production of prenylnaringenins in *S. cerevisiae* requires identifying a microbial PT that can catalyze the prenylation of naringenin, given that the only known naringenin PTs are the botanical SfN8DT-1 (Sasaki et al., 2008) and SfFPT (Chen et al., 2013) proteins. To demonstrate the utility of our naringenin biosynthesis platform yeast for prenylnaringenin production, a regiospecific botanical PTs was expressed in the YPNG006 yeast strain. Specifically, SfN8DT-1 (Sasaki et al., 2008), a protein derived from the plant *S. flavescens*, was used as the botanical PT; SfN8DT-1 has been shown to function as a naringenin PT by catalyzing the C-8 prenylation of naringenin in the source plant (Li et al., 2015; Sasaki et al, 2008, 2009).

**Fig. 3:**
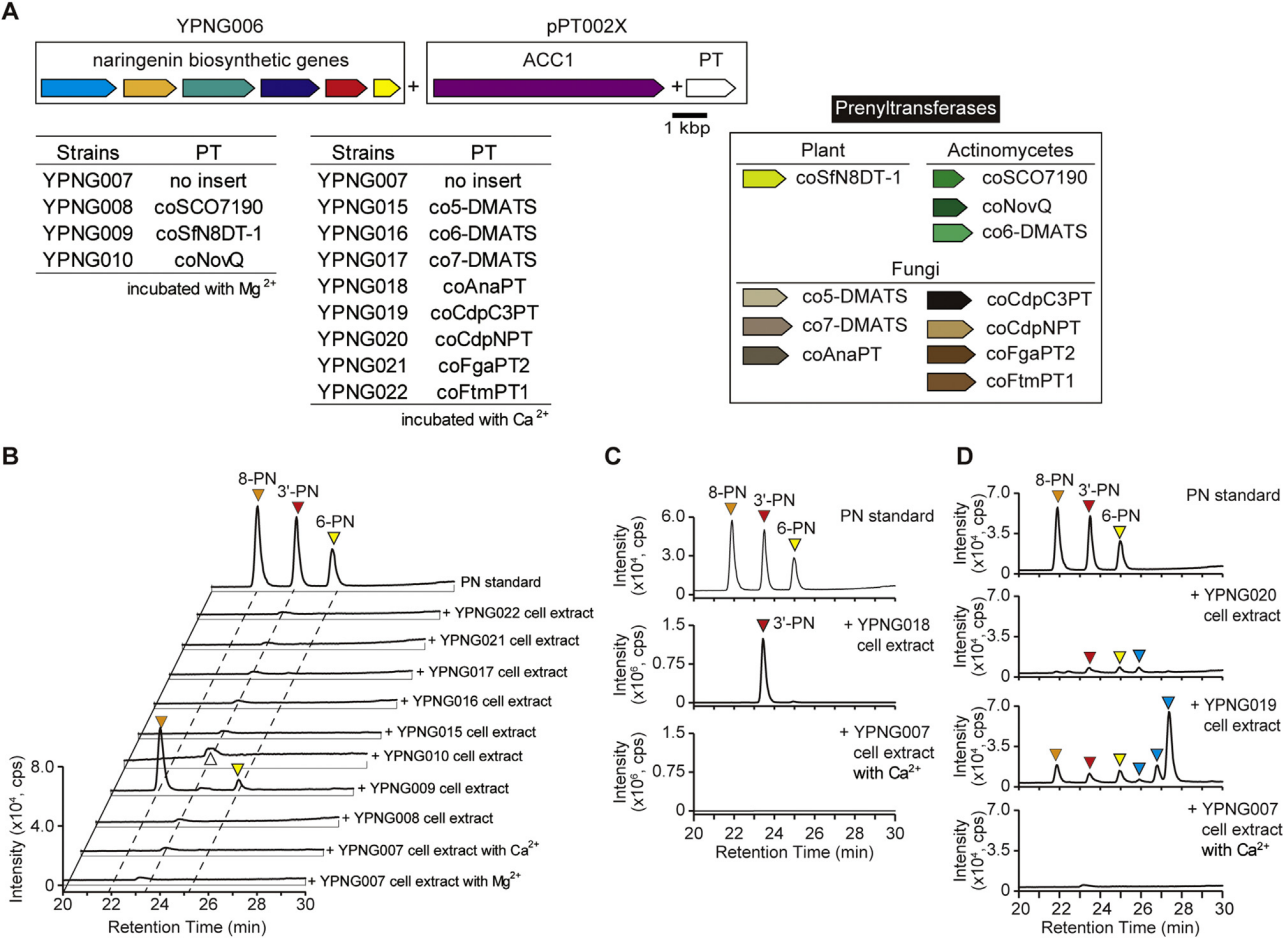
*In vitro* prenylation assay for naringenin using various prenyltransferases (PTs) expressed in yeast. Engineered yeasts were cultured overnight in YPDA rich medium, and cells then were disrupted for *in vitro* assays using expressed enzymes. Crude cell lysates were mixed with 0.2 ​mM naringenin, 0.2 ​mM DMAPP, and divalent ions (5 ​mM MgCl_2_ for YPNG008-010 or 10 ​mM CaCl_2_ for YPNG015-022); the reaction mixtures then were incubated at 30 ​°C for 6 ​h. The reaction products were extracted and analyzed by LC-MS. (A) Schematic illustration of genes (colored arrows) and yeast strains used for prenylation assays. These strains were based on YPNG006 (naringenin biosynthesis platform strain with genomically integrated plasmids), but additionally include the yeast *ACC1* gene and various PT genes. (B) LC-MS analyses of reaction products. MS chromatograms of *m/z* 339.2 are illustrated. Botanical and actinomycete PTs were incubated with MgCl_2_; fungal PTs were incubated with CaCl_2_. A peak in the MS chromatogram of the YPNG010 reaction products (indicated by a white triangle) exhibited a retention time similar to that of 3′-PN, but the MS spectrum of this peak was distinct from those of known prenylnaringenins. 8-PN and 6-PN were detected in the YPNG009 reaction product. Right axis indicates the intensity for reaction products of the YPNG018 ​cell extract; left axis indicates the intensity for reaction products of the other strains. (C) LC-MS analyses of the reaction products of YPNG018 (expressing coAnaPT) and YPNG007 (negative control) cell extracts incubated with naringenin and Ca^2+^. MS chromatograms of *m/z* 339.2 are illustrated. The YPNG018 ​cell extract catalyzed the C-3′ prenylation of naringenin, producing 3′-PN. (D) LC-MS analysis of reaction products of YPNG019 (expressing coCdpC3PT), YPNG020 (coCdpNPT), and YPNG007 (negative control) cell extracts incubated with naringenin and Ca^2+^. MS chromatograms of *m/z* 339.2 are illustrated. The YPNG019 ​cell extract catalyzed the production of 8-PN, 3′-PN, and 6-PN; the YPNG020 ​cell extract catalyzed the production of 3′-PN and 6-PN. Three other putative (but unidentified) prenylated naringenins also were detected in the YPNG019 reaction products, while one such putative (but unidentified) prenylated naringenin was detected in the YPNG020 reaction products (indicated by blue triangles).

A codon-optimized SfN8DT-1 gene (*coSfN8DT-1*) was synthesized and subcloned into a genome-integration vector. To increase the malonyl-CoA supply, which is necessary for the CHS (chalcone synthase) reaction (Fig. 1), the yeast *ACC1* gene, which encodes the endogenous acetyl-CoA carboxylase (ACC) that catalyzes acetyl-CoA carboxylation to generate malonyl-CoA (Roggenkamp et al., 1980), was concurrently cloned and inserted into the same plasmid (Table 1 and Supplementary Fig. S2). The resulting plasmid (pPT0022) then was introduced into YPNG006 (resulting in strain YPNG009) (Fig. 3A and Table 2).

Before testing this strain in an *in vivo* production experiment, the cells were used for an *in vitro* enzyme reaction. Harvested YPNG007 (negative control) (Table 2) and YPNG009 (*coSfN8DT-1*) cells were disrupted, and the resulting cell lysates were incubated with naringenin, DMAPP, and Mg^2+^, all of which are factors necessary for the prenylation reaction. The reaction products were extracted and analyzed by LC-MS. Incubation of these factors with the YPNG009 ​cell extract resulted in the synthesis of 8-PN, along with much smaller peak that matched the molecular mass and the retention time with 6-PN (Fig. 3B).

**Fig. 4:**
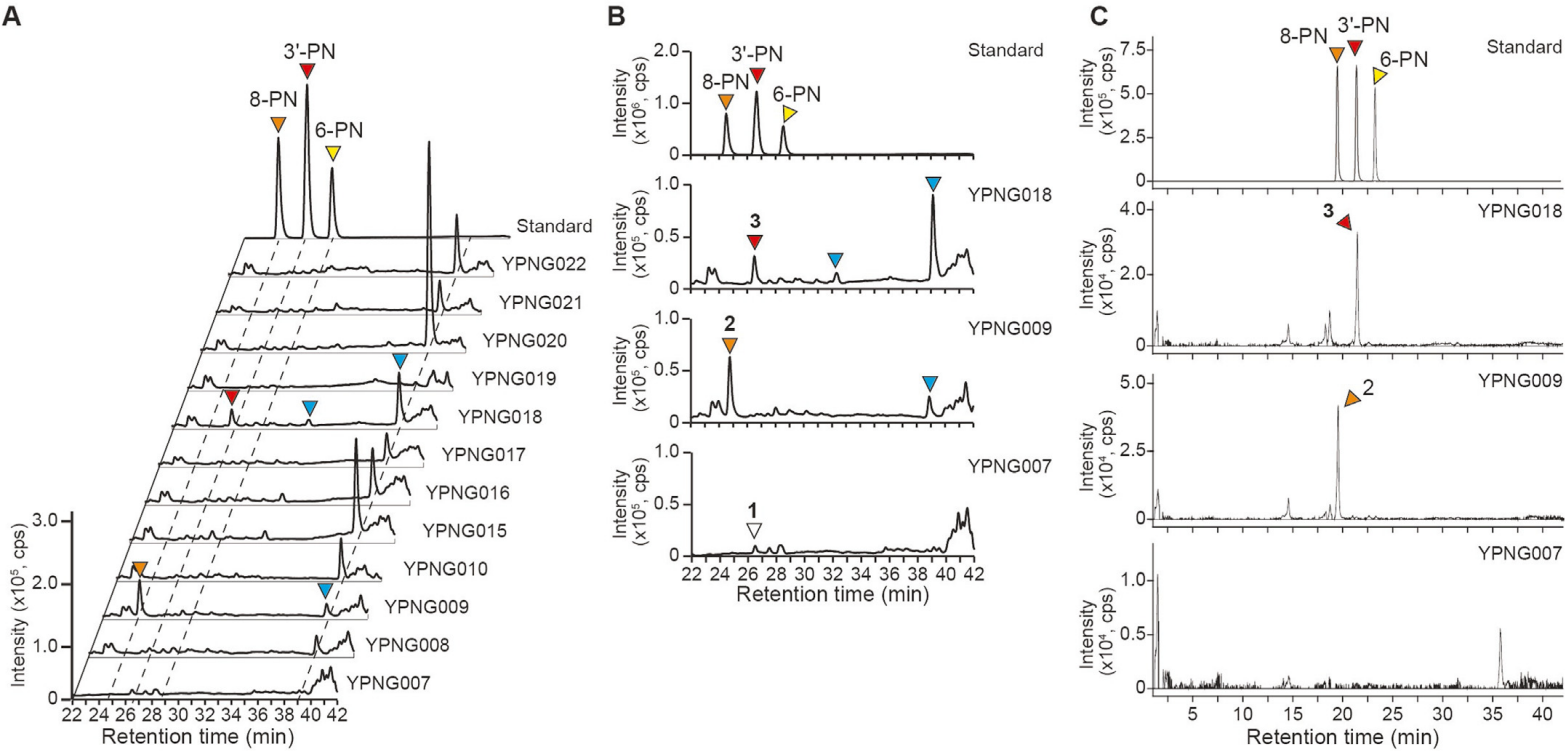
Metabolite analysis of prenylnaringenin-producing yeasts. (A, B) LC-MS analysis of metabolites produced by prenylnaringenin-producing yeasts. MS chromatograms of *m/z* 339.2 ​± ​0.6 are illustrated. YPNG009 and YPNG018 showed the same peaks with 8-PN (orange triangle; peak 2) and 3′-PN (red triangle; peak 3) standards, respectively. Two putative prenylated compounds were also detected (indicated by blue triangles). The MS spectrum of peak 1 (white triangle) was distinct from those of known prenylnaringenins. (C) LC-QTOF/MS analysis of metabolites produced by YPNG009 and YPNG018. MS chromatograms of *m/z* 339.124 ​± ​0.002 are illustrated. YPNG009 and YPNG018 showed the same peaks with 8-PN (orange triangle; peak 2) and 3′-PN (red triangle; peak 3) standards, respectively. Two putative prenylated compounds (indicated by blue triangles in Fig. 4A and B) were not detected in the chromatogram with the narrow mass window (±0.002). The MS/MS spectra from the precursor ions of peak 2 (orange triangle) and peak 3 (red triangle) were well consistent with those of 8-PN and 3′-PN standards, respectively (Fig. S4). On the other hand, the MS/MS spectra obtained from the precursor ions of the putative PN compounds (blue triangles) were not similar to those of any PN standards, in addition to the difference of exact *m/z* of the precursor ions (339.199) (Fig. S4).

Subsequently, an *in vivo* prenylnaringenin production experiment was performed using the same YPNG007 and YPNG009 yeast strains. The cells were cultured in YPDA rich medium containing 10 ​mM ʟ-phenylalanine, and the produced metabolites were analyzed by LC-MS. Consistent with our expectation, YPNG009 demonstrated the reasonably selective heterologous production of 8-PN from phenylalanine; the strain possibly might have also produced another putative prenylated compounds (Fig. 4A and B).

### 3.4 In vitro screening of microbial prenyltransferases for prenylnaringenin production

To screen promiscuous microbial PTs for their function in yeast, an *in vitro* naringenin prenylation assay was conducted. Eleven promiscuous PTs were selected from among actinomycete or fungal secondary metabolite biosynthetic enzymes (Table 3). These microbial PTs belong to the ABBA prenyltransferase superfamily, which can be classified into the two families, the CloQ/NphB family and the DMATS/CymD family. These families are known to correspond to phenol/phenazine PTs and indole PTs, respectively (Bonitz et al., 2011). Within the CloQ/NphB family, the bacterial (actinomycete) PTs (dimethylallyltransferases) SCO7190(Kumano et al., 2008) and NovQ (Ozaki et al., 2009) are known to catalyze the prenylation of phenylpyruvate. Within the DMATS/CymD family, 5-DMATS (Yu et al., 2012), 7-DMATS (Kremer et al., 2007), AnaPT (Yin et al., 2009), CdpC3PT (Yin et al., 2010), CdpNPT (Yin et al., 2007), FgaPT2 (Unsöld and Li, 2005), and FtmPT1 (Grundmann and Li, 2005) are fungal PTs that serve as tryptophan dimethylallyltransferases (or dimethylallyl tryptophan synthases; DMATSs), while 6-DMATS (Winkelblech and Li, 2014) is an actinomycete PT.

**Table 3:** Candidate prenyltransferases for naringenin prenylation. Eleven promiscuous microbial prenyltransferases (PTs) were selected for testing for prenylation of naringenin in yeast. A botanical naringenin PT, SfN8DT-1, was used as the positive control.

Source	Name	Species	*in vitro* Prenylation activity to flavonoids	Plenylation position of Naringenin	Reference
**Plant**	SfN8DT-1	*Sophora flavescens*	Naringenin (physiological substrate), Liquiritigenin, Hesperetin	C-8 of A-ring	Levisson et al. (2019), Li et al. (2015), Sasaki et al. (2008, 2009)
**Actinomycetes**	SCO7190	*Streptomyces coelicolor* A3 (2)	Naringenin	C-6 of A-ring	Kumano et al. (2008)
	NovQ	*Actinoalloteichus cyanogriseus* (*Streptomyces niveus*)	Naringenin, Apigenin, Daizein, Genistein	C-3′ and 4-O′ of B-ring	Ozaki et al. (2009)
	6-DMATS	*Streptomyces ambofaciens*	–	–	Winkelblech and Li (2014)
**Fungi**	5-DMATS	*Aspergillus clavatus*	–	–	Yu et al. (2012)
	7-DMATS	*Aspergillus fumigatus*	Naringenin, 7-hydroxyflavanone, Eriodictyol, Hesperetin, Silibinin, Phloretin, Apigenin, Genistein, Biochanin A	C-6 of A-ring	Kremer et al. (2007), Zhou et al. (2015)
	AnaPT	*Neosartorya fischeri* (*Aspergillus fischeri*)		C-3′ of A-ring	Yin et al. (2009), Zhou et al. (2015)
	CdpC3PT	*N. fischeri* (*A. fischeri*)	–	–	Yin et al. (2010)
	CdpNPT	*A. fumigatus*	–	–	Yin et al. (2007)
	FgaPT2	*A. fumigatus*	–	–	Unsöld and Li (2005)
	FtmPT1	*A. fumigatus*	–	–	Grundmann and Li (2005)

Among these microbial PTs, SCO7190, NovQ, 7-DMATS, and AnaPT have been shown to be able to catalyze the prenylation of naringenin *in vitro*, either at the C-6 of the A-ring (SCO7190(Kumano et al., 2008) and 7-DMATS (Zhou et al., 2015)), the C-3′ and O-4′ of B-ring (NovQ (Ozaki et al., 2009)), or the C-3’ of the B-ring (AnaPT (Zhou et al., 2015)) (Table 3). Although other microbial PTs of the DMATS group have not been reported to catalyze the prenylation of naringenin, some of these enzymes have been shown to accept the naphthalene backbone as a substrate, consistent with their relaxed substrate specificity (Yu et al., 2011). Therefore, these actinomycete and fungal PTs were selected as candidate enzymes to test the potential for transferring a prenyl group to naringenin when expressed in yeast.

These eleven microbial PT genes were codon optimized and subcloned into a genome integration vector along with the *ACC1* gene (Table 1 and Supplementary Fig. S2); the resulting plasmids were designated pPT0021, pPT0023–pPT0029, pPT002A, and pPT002B. Each of these genome integration plasmids then was introduced into YPNG006 (the naringenin-producing yeast platform strain) (Fig. 3A), yielding strains YPNG008, YPNG010, and YPNG015–022). First, *in vitro* enzyme reactions were conducted (Fig. 3B). Cultured YPNG007 (negative control lacking PT), −008, −010, and −015 to −022 ​cells were harvested and disrupted, and cell lysates were incubated with naringenin, DMAPP, and Mg^2+^ or Ca^2+^ (cofactors that are necessary for PT activity). The reaction products were extracted and analyzed by LC-MS.

The YPNG018 (harboring *coAnaPT*) cell extract catalyzed C-3′ prenylation of naringenin, providing the production of 3′-PN, as predicted from a previous *in vitro* study (Zhou et al., 2015) (Fig. 3C). YPNG019 (harboring *coCdpC3PT*) cell extract showed a mixture of peaks consistent with 8-PN, 3′-PN, and 6-PN (Fig. 3D). In contrast, the YPNG020 (harboring *coCdpNPT*) cell extract showed a mixture of peaks consistent with 3′-PN and 6-PN (Fig. 3D). Interestingly, additional putative prenylated compounds (distinct from the known PNs, and presumed to be modifications of naringenin or (unlikely but) of other endogenous compounds with the same molecular mass) also were detected in the reaction products of the YPNG019 and YPNG020 ​cell extracts (Fig. 3D). Thus, these three microbial PTs were shown to be able to catalyze the prenylation of naringenin *in vitro*, although other PTs may also have the potential to produce the PNs if we can find the optimal reaction conditions.

### 3.5 Heterologous production of 3′-prenylnaringenin in yeast using promiscuous microbial prenyltransferases

In order to confirm the production of prenylnaringenins by YPNG008-010 and −015 to −022 *in vivo*, these strains were grown in YPDA rich medium containing 10 ​mM ʟ-phenylalanine; the produced metabolites then were analyzed by LC-MS. Fig. 4A shows the analytical data for the prenylated products. YPNG018 (harboring *coAnaPT*) produced 3′-PN from phenylalanine *in vivo*, consistent with outcome of the *in vitro* assay (Fig. 4B). This result was the first demonstration (to our knowledge) of the *in vivo* heterologous production of 3′-PN in a microorganism. In contrast to our *in vitro* results, YPNG019 (harboring *coCdpC3PT*) and YPNG020 (harboring *coCdpNPT*) synthesized none of the well-known prenylnaringenins (8-PN, 3′-PN, and 6-PN), but almost every prenyltransferase-introducing yeast including YPNG009 and YPNG018 (8-PN and 3′-PN producing strains, respectively) produced one or two putative prenylated compounds of unknown identity (Fig. 4A and B). To identify whether these compounds are prenylated naringenin or not, the metabolites produced by YPNG009 and YPNG018 were further analyzed by LC-QTOF/MS. The MS chromatograms of LC-QTOF/MS analysis at *m/z* 339.124 ​± ​0.002 (*m/z* 339.2 ​± ​0.6 was used for LC-MS analysis) demonstrated that YPNG009 and YPNG018 produced the compounds with the same accurate *m/z* as 8-PN and 3′-PN, respectively (Fig. 4C). Additionally, the MS/MS spectra were well consistent with 8-PN and 3′-PN standards (Fig. S4). The putative prenylated compounds were not detected at *m/z* 339.124 ​± ​0.002 (Fig. 4C). These results indicate that YPNG009 and YPNG018 surely produced 8-PN and 3′-PN, respectively, and the unknown peaks were not PNs.

The amount of 8-PN produced by YPNG009 (harboring botanical *coSfN8DT-1*) was 0.119 ​± ​0.0345 ​μg/g DCW, while the amount of 3′-PN produced by YPNG018 (harboring microbial *coAnaPT*) was 0.196 ​± ​0.0147 ​μg/g DCW (Table 4). These yields also could be expressed as 0.615 ​± ​0.157 ​μg/L for 8-PN and 1.10 ​± ​0.0962 ​μg/L for 3′-PN (Table 4). Supplementary Fig. S5 shows the amount of consumed substrate (l-phenylalanine), byproduct (2-phenylethanol), and intermediates (*trans*-cinnamic acid, *p*-coumaric acid, and naringenin) in the various strains. Naringenin productivity of YPNG009 and YPNG018 (Supplementary Fig. S5C) was greatly increased compared with YPNG006 (Fig. 2C). Since YPNG009 and YPNG018 additionally expressed *ACC1* and PT genes in YPNG006, the increase of naringenin was probably attributed to the overexpression of *ACC1*, which is involved in the supply of malonyl-CoA. The initial concentration of l-phenylalanine was 2230 ​mg ​L^−1^, of which 70% was consumed by YPNG007, whereas approximately one third of the l-Phe was consumed by YPNG009 and YPNG018. A portion of the l-phenylalanine was converted to 2-phenylethanol by yeast endogenous enzymes, representing the production of 110 ​mg ​L^−1^ of 2-phenylethanol by YPNG007. On the other hand, the amount of 2-phenylethanol was nominally lower in YPNG009 and YPNG018 than in YPNG007. This result indicated that exogenous introduction of the naringenin biosynthesis pathway shifted the carbon flux from synthesis of 2-phenylethanol toward production of prenylnaringenin (naringenin), although the amounts of (prenyl)naringenin and intermediates produced were relatively low compared with the amount of l-phenylalanine consumed. This result suggested that most of the consumed l-phenylalanine might be used for cell growth or other by-products, and further improvement of (prenyl)naringenin production would be needed for future development.

**Table 4:** Amounts of produced prenylnaringenins. The amounts of prenylnaringenins produced by the engineered yeasts are indicated. Data are presented as the mean ​± ​SD of four independent transformants.

Strain	Product	Productivity (μg/g DCW)	Titer (μg/l)
YPNG009	8-prenylnaringenin (8-PN)	0.119 ​± ​0.0345	0.615 ​± ​0.157
YPNG018	3′-prenylnaringenin (3′-PN)	0.196 ​± ​0.0147	1.10 ​± ​0.0962

## 4 Discussion

Our prenylnaringenin-producing yeast system revealed that the promiscuous microbial PTs can replace regiospecific botanical PTs. This result is especially interesting, given that most of botanical PTs still remain unidentified. To the best of our knowledge, this work is the first demonstration of first microbial production of 3′-PN, and was achieved by using the promiscuous fungal PT AnaPT (Yin et al., 2009) in combination with genes encoding components of the Arabidopsis naringenin biosynthetic pathway. Cell extracts of YPNG019 (harboring *coCdpC3PT*) and YPNG020 (harboring *coCdpNPT*) also catalyzed the prenylation of naringenin *in vitro*. The reaction products obtained using these fungal PTs included 8-PN, 3′-PN, and/or 6-PN, and three putative but unidentified prenylated compounds (whether naringenins or other molecules) (Fig. 3D). These results represent the first demonstration that CdpC3PT and CdpNPT, at least, can catalyze the prenylation of naringenin *in vitro*. Although YPNG019 and YPNG020 did not produce well-known prenylnaringenins *in vivo*, an increase in the level of the naringenin precursor might permit detection of the synthesis of these prenylnaringenins. These results suggest that the promiscuous microbial PTs used in this study have great potential for the production of prenylnaringenins or other prenylated compounds, possibly expanding the available positions on the flavonoid backbone that can be modified by the prenyl moiety.

The naringenin-producing yeast system expressing botanical SfN8DT-1 also produced 8-PN from l-phenylalanine. This study used l-phenylalanine as a substrate, which is the initial compound of flavonoid biosynthesis. Notably, l-Phe is simpler, less expensive, and more water-soluble than are the substrates (naringenin (Sasaki et al., 2009) and *p*-coumaric acid (Li et al., 2015)) used in previous studies. Recently, the production of 8-PN from glucose was reported (Levisson et al., 2019). Compared with that report, our yeast production system showed low productivity of naringenin from l-phenylalanine and much lower bioconversion efficiency to prenylnaringenins; increasing the naringenin productivity of our system will be an important future challenge. Our metabolite analysis revealed the low productivity of the desired biological intermediates from l-phenylalanine, as indicated by comparison of l-phenylalanine consumption (~700 ​mg ​L^−1^) with the amounts of *trans*-cinnamic acid, *p*-coumaric acid, and naringenin generated (~2 ​mg ​L^−1^ each). These data suggest that the majority of the consumed l-phenylalanine is used for cell growth and/or other processes. Engineering of the host yeast strain is expected to improve the productivity, as has been demonstrated in previous reports (Koopman et al., 2012; Levisson et al., 2019). The low biosynthetic rate also may be caused by the insufficient expression of the biosynthetic enzymes. Codon optimization is one way of increasing the expression level of enzymes. Previous studies have demonstrated that the codon optimization of foreign gene(s) significantly increases the production of compounds (Ajikumar et al., 2010) or proteins (Norkiene and Gedvilaite, 2012), given that codon optimality contributes to translation efficiency and mRNA stability (Presnyak et al., 2015). Indeed, codon optimization of the *AtCHS3* gene considerably increased naringenin production in the present study (Fig. 2). In addition, the screening of gene sources other than *A. thaliana* would be also effective for increasing the naringenin productivity (Mark et al., 2019).

The amounts of produced prenylnaringenins reveal the low conversion efficiency of naringenin (~2 ​mg ​L^−1^) to 8-PN and 3′-PN (~1 ​μg/L). These data indicated that the PTs used here may have low prenylation activity for naringenin (Zhou et al., 2015) and/or that the biosynthesis of prenylnaringenins may be limited by an insufficient supply of substrates. A previous investigation of 8-PN production in *S. cerevisiae* demonstrated that the low availability of DMAPP impaired 8-PN production; yeast engineered to have a larger DMAPP pool exhibited increased 8-PN production (Li et al., 2015). Therefore, increasing the availability of DMAPP is expected to improve the productivity of prenylnaringenins. For example, additional expression of the rate-limiting enzyme of the mevalonate (MVA) pathway, 3-hydroxy-3-methylglutaryl-coenzyme A reductase (HMGR), might be an effective metabolic engineering strategy for increasing the pool of available DMAPP(Polakowski et al., 1998) in our strains. Therefore, the truncated *HMG1* (*tHMG1*) (Polakowski et al., 1998) and the acetoacetyl-CoA synthase gene (*nphT7*) from *Streptomyces* (Okamura et al., 2010) were additionally co-expressed in YPNG009 (8-PN producer); however, we did not observe the increase of 8-PN productivity (data not shown). Thus, other strategy would be needed to improve the DMAPP supply for increasing the productivity of prenylnaringenin.

This study demonstrated that promiscuous microbial PTs have great potential for use in the production of various prenylated naringenins and other molecules, including unnatural compounds, although these microbial PTs displayed low regiospecificity for prenyl group transfer (Fig. 3D). The crystal structures of several ABBA PTs have been elucidated (Chen et al., 2016; Fan et al., 2015; Jost et al. (2010); Kuzuyama et al., 2005; Liu et al., 2013; Metzger et al., 2010; 2009; Mori et al., 2016), and mutagenesis of these enzymes has been shown to alter the prenyl donor selectivity and regiospecificity of prenylation (Chen et al., 2016; Fan et al., 2015; Mori et al., 2016). Therefore, on-target production of various prenylnaringenins should be possible by protein engineering of promiscuous microbial PTs to enhance their prenylation selectivity.

**Fig. 5:**
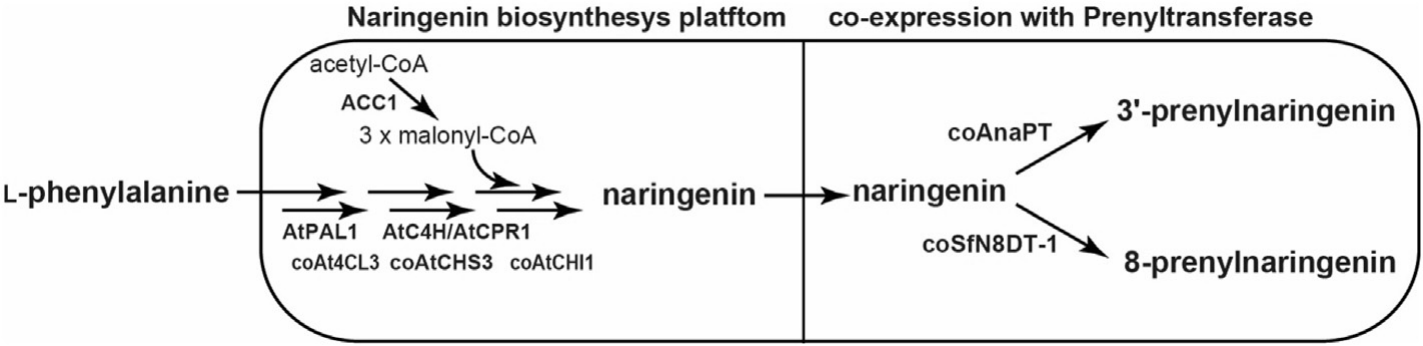
Production of prenylnaringenin by expressing promiscuous microbial PTs in yeast. A schematic summary of this paper. An engineered yeast strain produced 3′-prenylnaringenin using a promiscuous microbial PT (AnaPT) in combination with the six naringenin biosynthetic enzymes.

In conclusion, we showed that promiscuous microbial PTs can be used to substitute for unidentified botanical PTs. We further demonstrated the first heterologous production of 3′-PN using AnaPT, a promiscuous fungal DMATS (Fig. 5). These PTs are expected to serve as useful tool for artificial biosynthesis of novel prenylflavonoids, compounds that are difficult to produce in nature. Furthermore, we also revealed that PTs with relaxed substrate specificity have great potential to provide a diversity of prenylated compounds, including both well-known and novel prenylnaringenins. These findings suggest strategies towards expanding the variety of prenylated compounds, as well as for providing substitutes for botanical PTs, most of which remain unidentified and are restricted by the relatively strict regiospecificity of their prenyl group transfer activities.

## Funding

This work was supported by the Commission for Development of Artificial Gene Synthesis Technology for Creating Innovative Biomaterials from the Ministry of Economy, Trade and Industry (METI), Japan. This work also was partially supported by Project P16009, Development of Production Techniques for Highly Functional Biomaterials Using Smart Cells of Plants and Other Organisms (Smart Cell Project) of the New Energy and Industrial Technology Development Organization (NEDO); and by the JST-Mirai Program (Grant Number JPMJMI17EJ), Japan.

## CRediT authorship contribution statement

**Shota Isogai:** Conceptualization, Data curation, Formal analysis, Investigation, Methodology, Resources, Validation, Visualization, Writing – original draft. **Nobuyuki Okahashi:** Data curation, Formal analysis, Investigation, Methodology. **Ririka Asama:** Data curation, Formal analysis, Methodology. **Tomomi Nakamura:** Data curation, Formal analysis, Methodology. **Tomohisa Hasunuma:** Investigation, Funding acquisition, Project administration, Supervision. **Fumio Matsuda:** Data curation, Formal analysis, Investigation, Methodology. **Jun Ishii:** Conceptualization, Investigation, Methodology, Resources, Funding acquisition, Project administration, Supervision, Writing – review & editing. **Akihiko Kondo:** Resources, Funding acquisition, Project administration, Supervision.

## Declaration of competing interest

We confirm that there are no conflicts of interest associated with this publication.
