# Supplementary material for: Synthetic production of prenylated naringenins in yeast using promiscuous microbial prenyltransferases
Source: Metab Eng Commun. 2021 Mar 19;12:e00169. doi: 10.1016/j.mec.2021.e00169 (PMC8040282; doi:10.1016/j.mec.2021.e00169)
Supplement: Multimedia component 2 [file mmc2.docx]

**Supplementary Data for**

**Synthetic production of prenylated naringenins in yeast using promiscuous microbial prenyltransferases**

**Shota Isogai^1,2^, Nobuyuki Okahashi^4^, Ririka Asama^1^, Tomomi Nakamura^1,2^,** **Tomohisa Hasunuma^1,2,3^, Fumio Matsuda^4^, Jun Ishii^1,2,3,*^,** **Akihiko Kondo^1,2,3,5,6,*^**

1. Graduate School of Science, Technology and Innovation, Kobe University, 1-1 Rokkodai, Nada, Kobe 657-8501, Japan
2. Technology Research Association of Highly Efficient Gene Design (TRAHED), Kobe, Japan
3. Engineering Biology Research Center, Kobe University, 1-1 Rokkodai, Nada, Kobe 657-8501, Japan
4. Department of Bioinformatic Engineering, Graduate School of Information Science and Technology, Osaka University, 1-5 Yamadaoka, Suita, Osaka 565-0871, Japan
5. Department of Chemical Science and Engineering, Graduate School of Engineering, Kobe University, 1-1 Rokkodai, Nada, Kobe 657-8501, Japan
6. Center for Sustainable Resource Science, RIKEN, 1-7-22 Suehiro, Tsurumi, Yokohama 230-0045, Japan

*Corresponding author: Jun Ishii

Engineering Biology Research Center, Kobe University, 1-1 Rokkodai, Nada, Kobe 657-8501, Japan

Tel: +81-78-803-6356; Fax: +81-78-803-6192; E-mail: junjun@port.kobe-u.ac.jp

*Corresponding author: Akihiko Kondo

Graduate School of Science, Technology and Innovation, Kobe University, 1-1 Rokkodai, Nada, Kobe 657-8501, Japan

Tel: +81-78-803-6196; Fax: +81-78-803-6196; E-mail: akondo@kobe-u.ac.jp


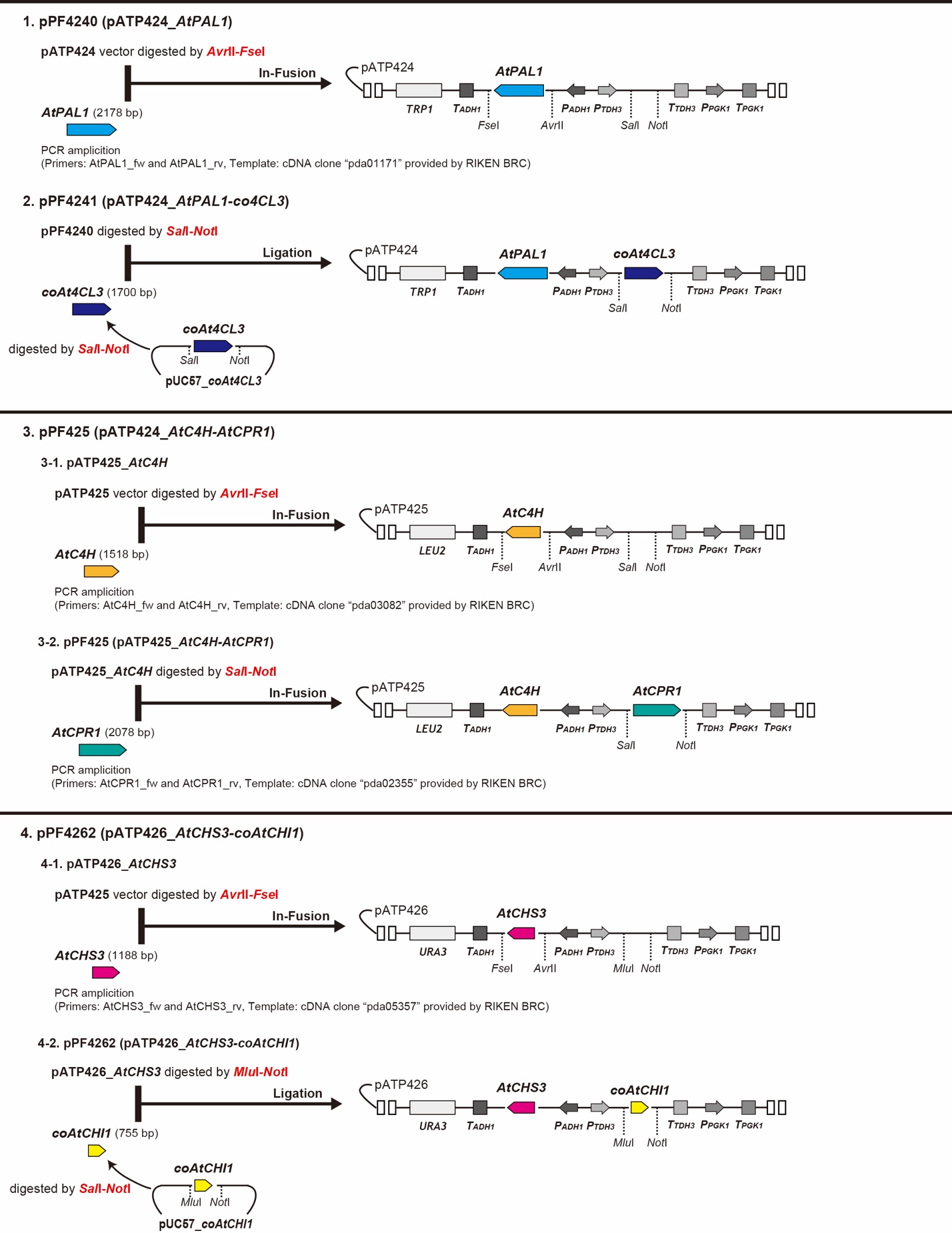


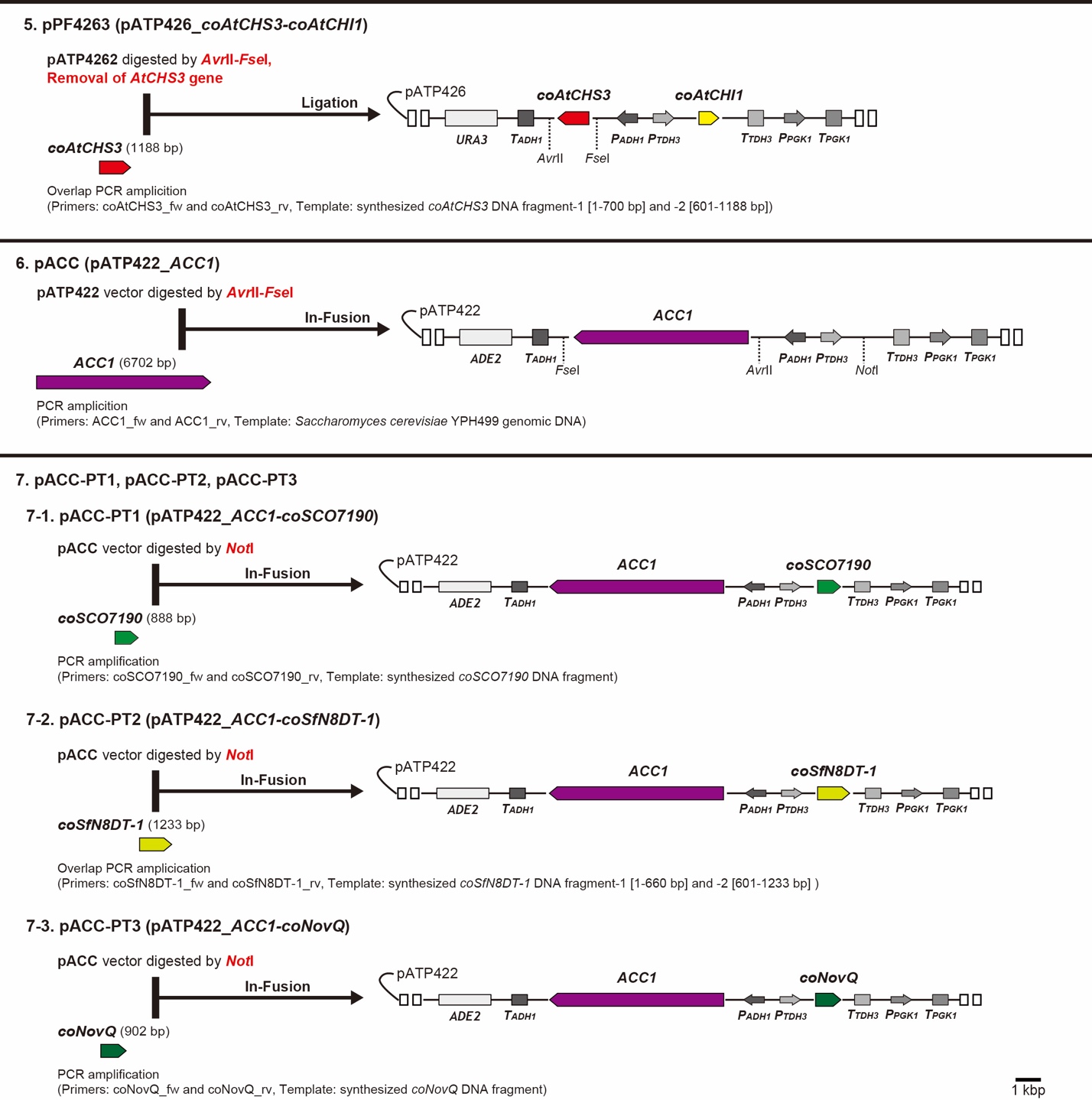


**Fig. S1. Schematic illustration of construction schemes for plasmids (multi-copy 2μ origin) used for expression of enzymes in the prenylnaringenin-producing strains.** A “co” prefix indicates “codon optimized”. The PCR amplification of genes was performed using primers (Table S2) and templates as described in the ***Materials and Methods***. The sizes of the colored arrows are proportional to the lengths of the corresponding genes or gene elements. pUC57_*coAt4CL3* and pUC57_*coAtCHI1* were synthesized using the GenScript Gene Synthesis service.


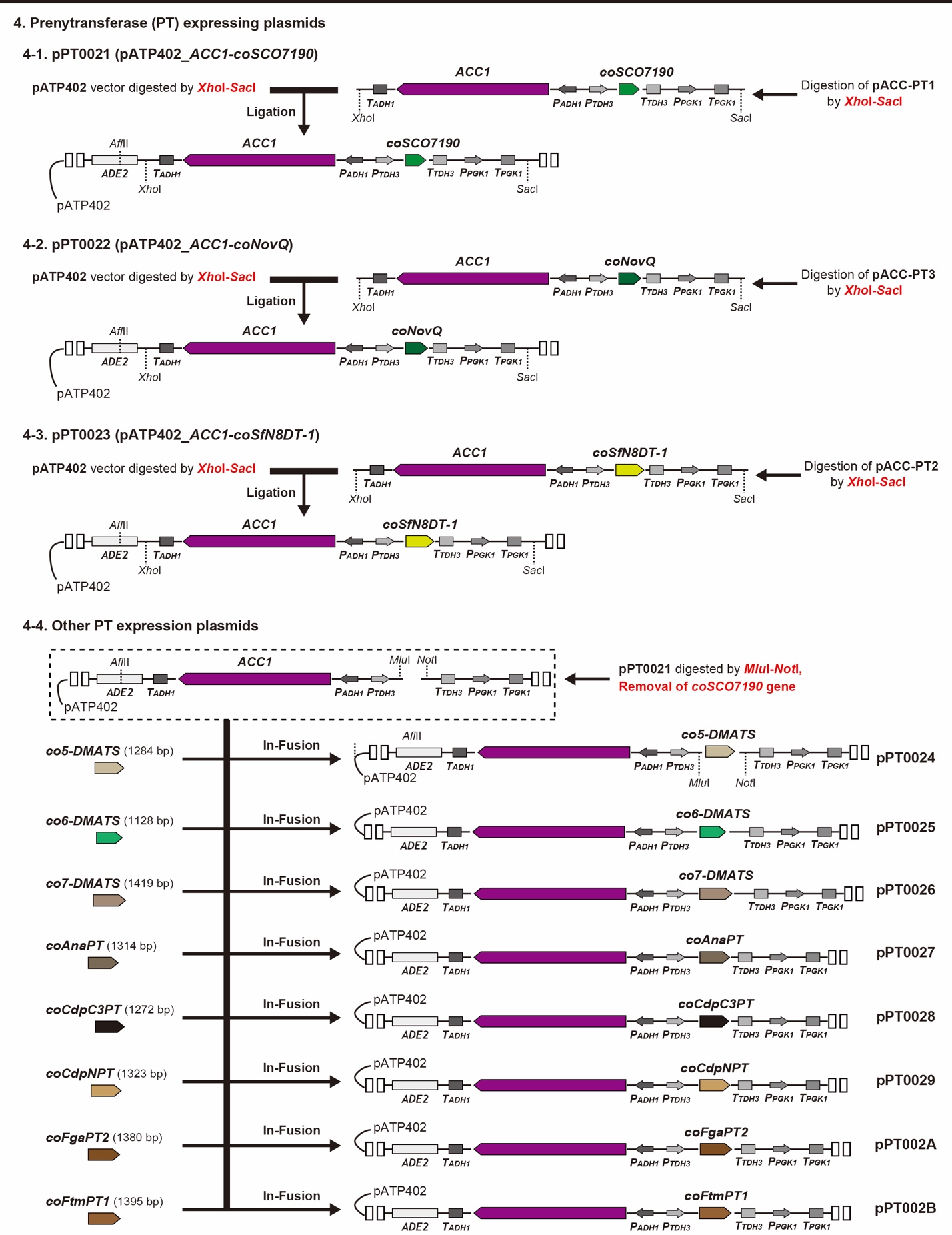

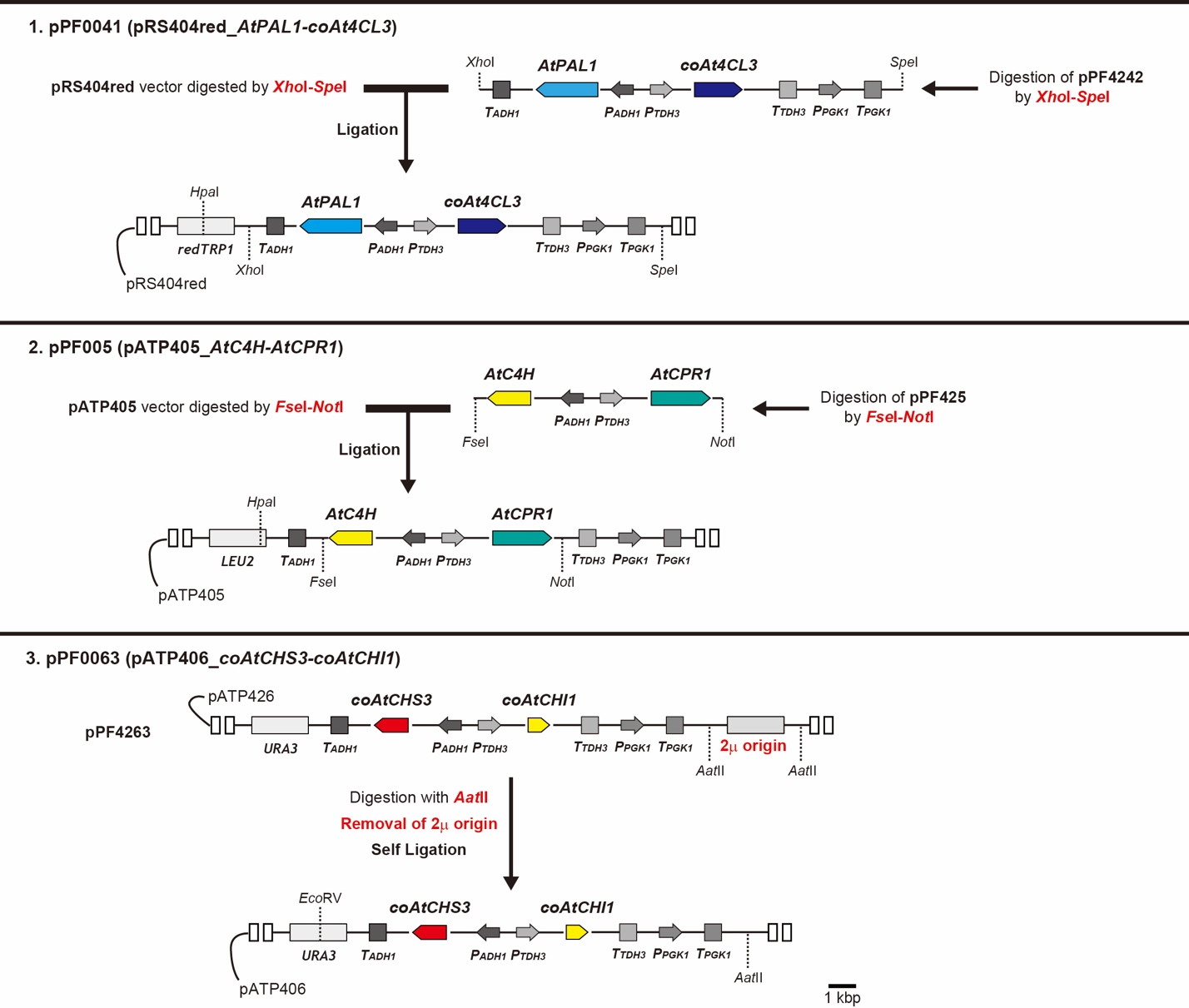


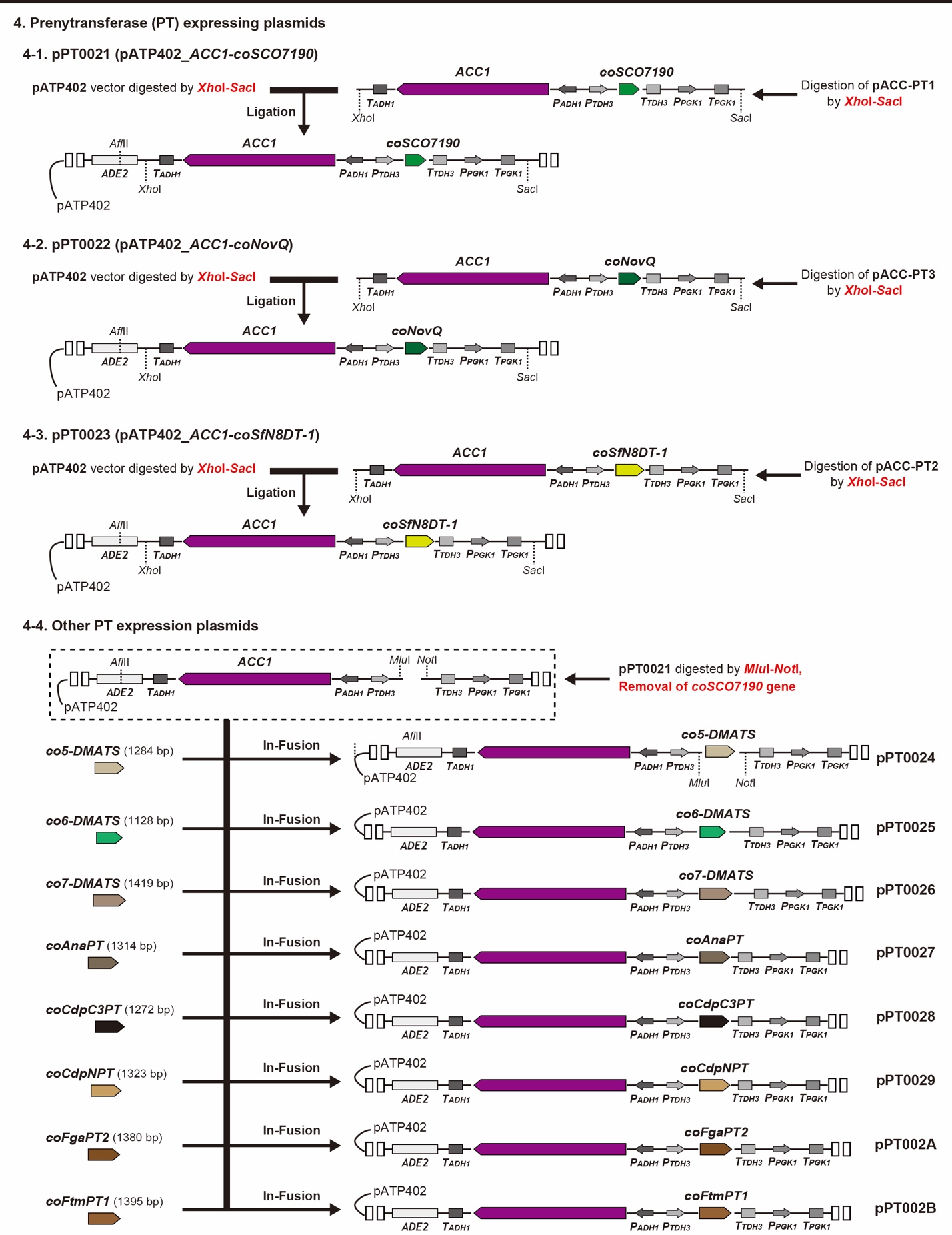


**Fig. S2. Schematic illustration of construction schemes for plasmids (genomic integration) used for expression of enzymes in the prenylnaringenin-producing strains.** A “co” prefix indicates “codon optimized”. The PCR amplification of PT genes was performed using primers (Table S2) and templates as described in the ***Materials and Methods***. The sizes of the colored arrows are proportional to the lengths of the corresponding genes or gene elements.


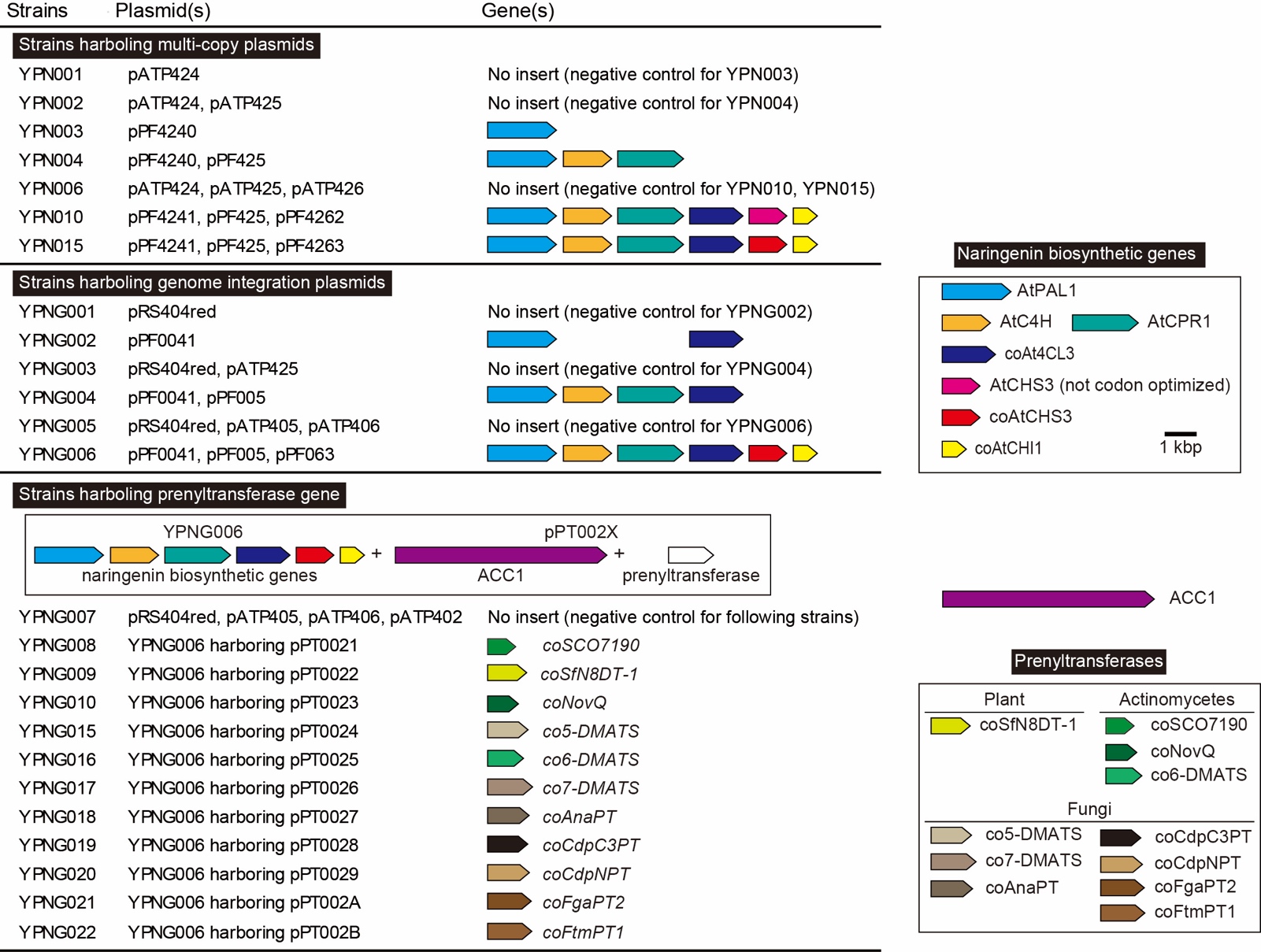


**Fig. S3. Schematic illustration of the strains constructed in this study.**


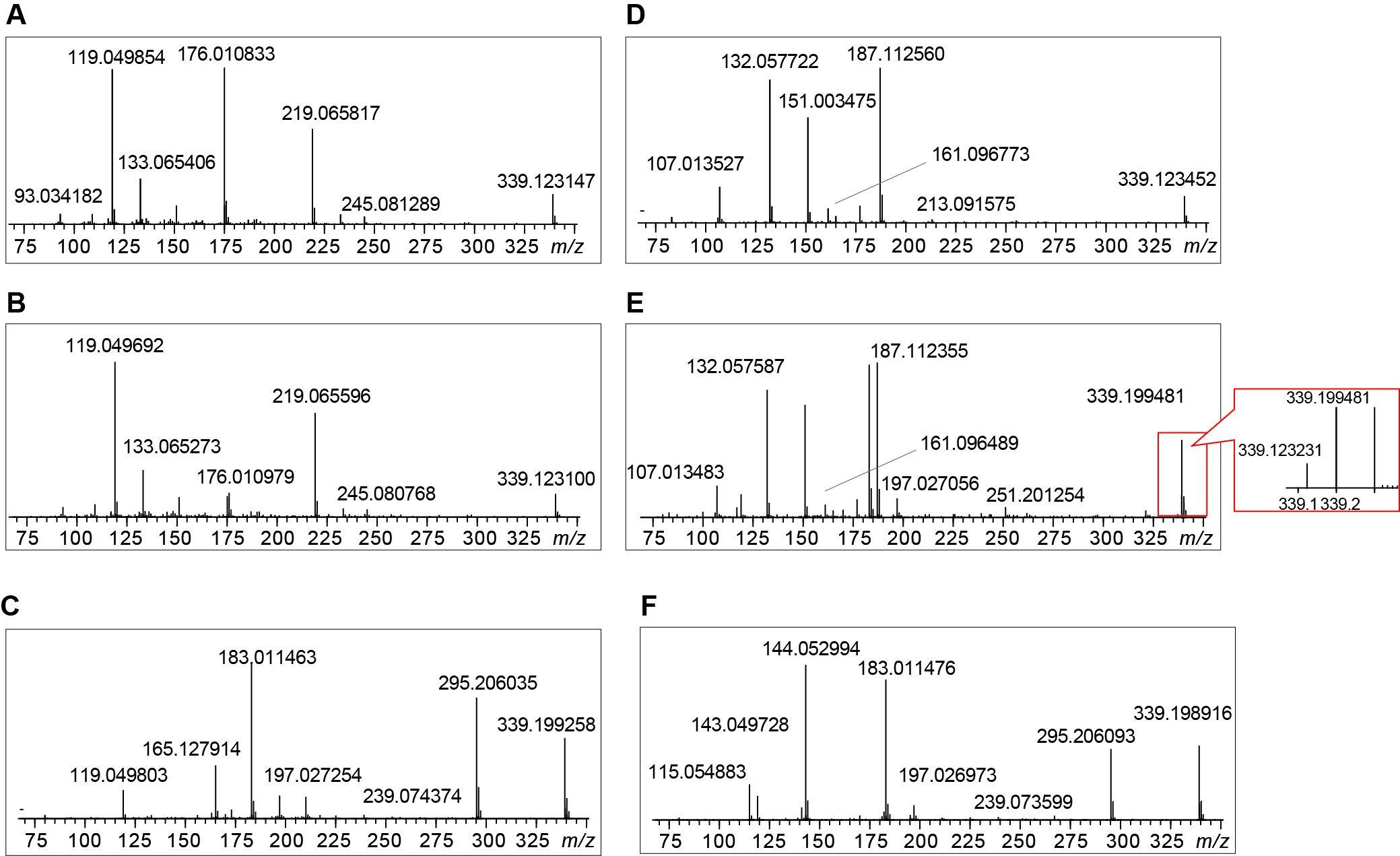


**Fig. S4. MS/MS spectra of PN standards and metabolites produced by YPNG009 and YPNG018.** MS/MS spectra of ions at m/z 339.124±1.5. (A) 8-PN standard, (B) peak 2 in YPNG009 metabolites (orange triangle in Fig. 4A and 4B), (C and F) putative prenylated compounds in YPNG009 and YPNG018 metabolites (Fig. 4A and 4B), (D) 3’-PN standard, and (E) peak 3 in YPNG018 metabolites (red triangle in Fig. 4A and 4B). The MS/MS spectra from the precursor ions of peak 2 and peak 3 were well consistent with 8-PN and 3’-PN standards, respectively. In contrast, the accurate m/z values (m/z 339.199) for the precursor ions of the putative prenylated compounds didn’t matched those of PNs (m/z 339.123).


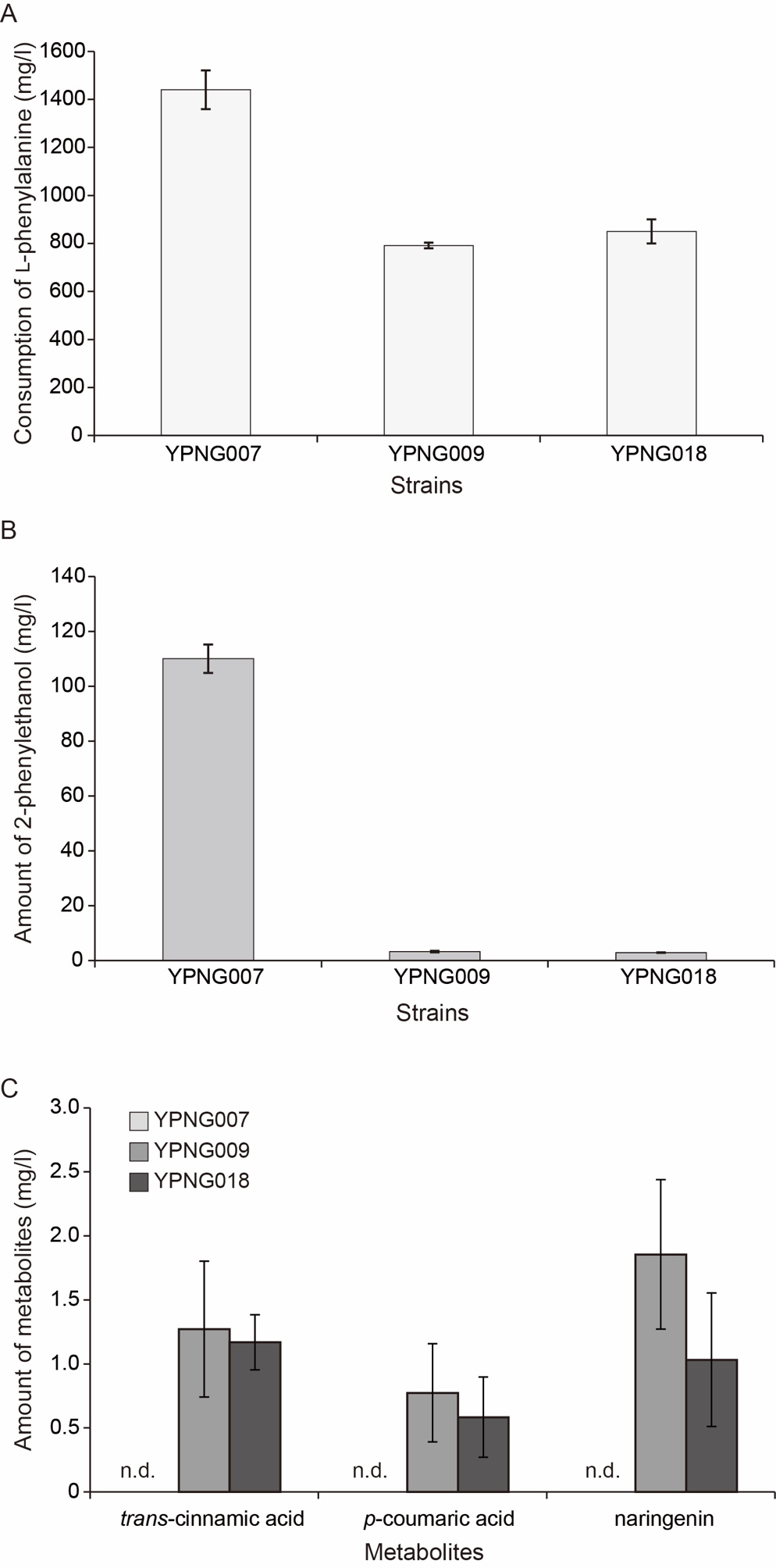


**Fig. S5. Metabolites produced by YPNG009 and YPNG018.** Engineered yeasts (assayed as four separate transformants each) were cultured in rich YPDA medium containing 10 mM l-phenylalanine for 96 hours; internal metabolites then were extracted and analyzed. The amounts of l-phenylalanine and 2-phenylethanol were analyzed by HPLC and those of naringenin and its intermediates (trans-cinnamic acid and *p*-coumaric acid) by LC-MS. Data are presented as mean ± SD of four independent transformants. (A) l-Phenylalanine consumption. Initial concentration of l-phenylalanine was 2230 mg/L in broth. The remaining l-phenylalanine was measured after 96 hours of cultivation and l-phenylalanine consumption was calculated. (B) Production of 2-phenylethanol. 2-Phenylethanol is synthesized from l-phenylalanine by endogenous yeast enzymes. (C) Comparison of biological intermediate production between YPNG009 and YPNG018. n.d. means “not detected”.

**Table S1. Genes used in this study**

| Name | Function of Gene Product | NCBI Accession | Reference |
| --- | --- | --- | --- |
| *ACC1* | acetyl-CoA carboxylase | CP020136 | Roggenkamp et al., (1980) |
| *AtPAL1* | phenylalanine ammonia lyase | AY045919 | Seki et al., (2002, 1998) |
| *AtC4H* | cinnamate-4-hydroxylase | AY065145 | Seki et al., (2002, 1998) |
| *AtCPR* | NADPH-ferrihemoprotein reductase ATR1 | AY054688 | Seki et al., (2002, 1998) |
| *At4CL3* | 4-coumarate CoA ligase | AY058083 | Seki et al., (2002, 1998) |
| *AtCHS3* | chalcone synthase | AY058155 | Seki et al., (2002, 1998) |
| *AtCHI1* | chalcone isomerase | BT004265 | Seki et al., (2002, 1998) |
|  | |  |  |
| **Botanical prenyltransferase (PT)** | |  |  |
| *SfN8DT-1* | naringenin 8-dimethlallyltransferase | AB325579 | Sasaki et al., (2008) |
|  | |  |  |
| **Actinomycetes PTs** | |  |  |
| *sco7190* | prenyltransferase | AB187170 | Kumano et al., (2008) |
| *novQ* | 4-hydroxyphenylpyruvate 3-dimethylallyltransferase | AB496950 | Ozaki et al., (2009) |
| *6-dmats* | dimethylallyltryptohane synthase | AM238663 | Winkelblech and Li, (2014) |
|  | |  |  |
| **Fungal PTs** | |  |  |
| *5-dmats* | dimethylallyltryptohane synthase | XM_001269816 | Yu et al., (2012) |
| *7-dmats* | 7-dimethylallyltryptohane synthase | EF539173 | Kremer et al., 2007), |
| *anaPT* | Indole diterpene prenyltransferase | XM_001258077 | Yin et al., (2009), |
| *cdpC3PT* | dimethylallyltryptohane synthase | NFIA_074280 | Yin et al., (2010) |
| *cdpNPT* | cyclic dipeptide N-prenyltransferase | EF433418 | Yin et al.,(2007) |
| *fgaPT2* | tryptophan dimethylallyltransferase | AY775787 | Unsöld and Li, (2005) |
| *ftmPT1* | brevianamide F prenyltransferase | AY861687 | Grundmann and Li, (2005) |

**Table S2. Primers used in this study**

| Target Gene | Primer | Restriction site | Sequence (5' to 3') |
| --- | --- | --- | --- |
| *ACC1* | Fw | *Avr*II | CTCATATACACCTAGGATGAGCGAAGAAAGCTTATT |
|  | Rv | *Fse*I | ATAAGAAATTCGCGGCCGGCCTTATTTCAAAGTCTTCAACAA |
|  |  |  |  |
| *AtPAL1* | Fw | *Avr*II | CTCATATACACCTAGGATGGAGATTAACGGGGCACACAA |
|  | Rv | *Fse*I | ATAAGAAATTCGCGGCCGGCCTTAACATATTGGAATGGGAGCT |
|  |  |  |  |
| *AtC4H* | Fw | *Avr*II | CTCATATACACCTAGGATGGACCTCCTCTTGCTGGAGAA |
|  | Rv | *Fse*I | ATAAGAAATTCGCGGCCGGCCTTAACAGTTCCTTGGTTTCA |
|  |  |  |  |
| *AtCPR1* | Fw | *Sal*I | AACAAACAAAGTCGACATGACTTCTGCTTTGTATGC |
|  | Rv | *Not*I | TAAATTCACGCGGCCGCTCACCAGACATCTCTGAGGTAT |
|  |  |  |  |
| *AtCHS3* | Fw | *Avr*II | CTCATATACACCTAGGATGGTGATGGCTGGTGCTTCT |
|  | Rv | *Fse*I | ATAAGAAATTCGCGGCCGGCCTTAGAGAGGAACGCTGTGCAA |
|  |  |  |  |
| *coAtCHS3* | Fw | *Avr*II | CTCATATACACCTAGGATGGTTATGGCTGGTGCTTCTTC |
|  | Rv | *Fse*I | ATAAGAAATTCGCGGCCGGCCTCACAATGGGACAGAATGCAAG |
|  |  |  |  |
| *coSCO7190* | Fw | *Not*I | GACACGCGTGCGGCCGCATGCCAACTGGTAGAACTAC |
|  | Rv | *Not*I | TAAATTCACGCGGCCGCTTATCTGTTCCAGTAACCAAAAAC |
|  |  |  |  |
| *coNovQ* | Fw | *Not*I | GACACGCGTGCGGCCGCATGCCAGCTTTGCCAATGAATC |
|  | Rv | *Not*I | TAAATTCACGCGGCCGCTTATCTAGCACCACCAGTAATAG |
|  |  |  |  |
| *coSfN8DT-1* | Fw | *Not*I | GACACGCGTGCGGCCGCATGGGTTCTATGTTGTTGGCTTC |
|  | Rv | *Not*I | TAAATTCACGCGGCCGCTTATCTGAACAAAGGGATCAAAAAG |
|  |  |  |  |
| *co5-DMATS* | Fw | *Mlu*I | CAAAGTCGACACGCGTATGCCACATCAAAAC |
|  | Rv | *Not*I | TAAATTCACGCGGCCGCTTACAACTTCCAGGA |
|  |  |  |  |
| *co6-DMATS* | Fw | *Mlu*I | CAAAGTCGACACGCGTATGACTACTGTTAGA |
|  | Rv | *Not*I | TAAATTCACGCGGCCGCTTATCTAACAGCAAC |
|  |  |  |  |
| *co7-DMATS* | Fw | *Mlu*I | CAAAGTCGACACGCGTATGTCCATTGGTGCC |
|  | Rv | *Not*I | TAAATTCACGCGGCCGCTTAGGTGGAGTAAAC |
|  |  |  |  |
| *coAnaPT* | Fw | *Mlu*I | CAAAGTCGACACGCGTATGTCCCCATTGTCT |
|  | Rv | *Not*I | TAAATTCACGCGGCCGCTTACAAGTTACCCTTC |
|  |  |  |  |
| *coCdpC3PT* | Fw | *Mlu*I | CAAAGTCGACACGCGTATGACCGTTTCTTCT |
|  | Rv | *Not*I | TAAATTCACGCGGCCGCTCAGTGGTAGTACATG |
|  |  |  |  |
| *coCdpNPT* | Fw | *Mlu*I | CAAAGTCGACACGCGTATGGATGGTGAAATG |
|  | Rv | *Not*I | TAAATTCACGCGGCCGCTCATTCAGGCCAGAAG |
|  |  |  |  |
| *coFgaPT2* | Fw | *Mlu*I | CAAAGTCGACACGCGTATGAAGGCTGCTAATG |
|  | Rv | *Not*I | TAAATTCACGCGGCCGCTTAATGTAAACCGGAG |
|  |  |  |  |
| *coFtmPT1* | Fw | *Mlu*I | CAAAGTCGACACGCGTATGCCACCAGCTCCAC |
|  | Rv | *Not*I | TAAATTCACGCGGCCGCTTAGTTTGGGAAAGAG |

Restriction sites are underlined. co: codon optimized
